# Supplementary material for: Comparative Genomics Reveals Genetic Diversity and Metabolic Potentials of the Genus Qipengyuania and Suggests Fifteen Novel Species
Source: Microbiol Spectr. 2022 Apr 21;10(3):e01264-21. doi: 10.1128/spectrum.01264-21 (PMC9241875; doi:10.1128/spectrum.01264-21)
Supplement: SUPPLEMENTAL FILE 1 — Supplemental material. Download spectrum.01264-21-s001.pdf, PDF file, 3.6 MB [file spectrum.01264-21-s001.pdf]

Supplementary Figures and Tables

Figure S1

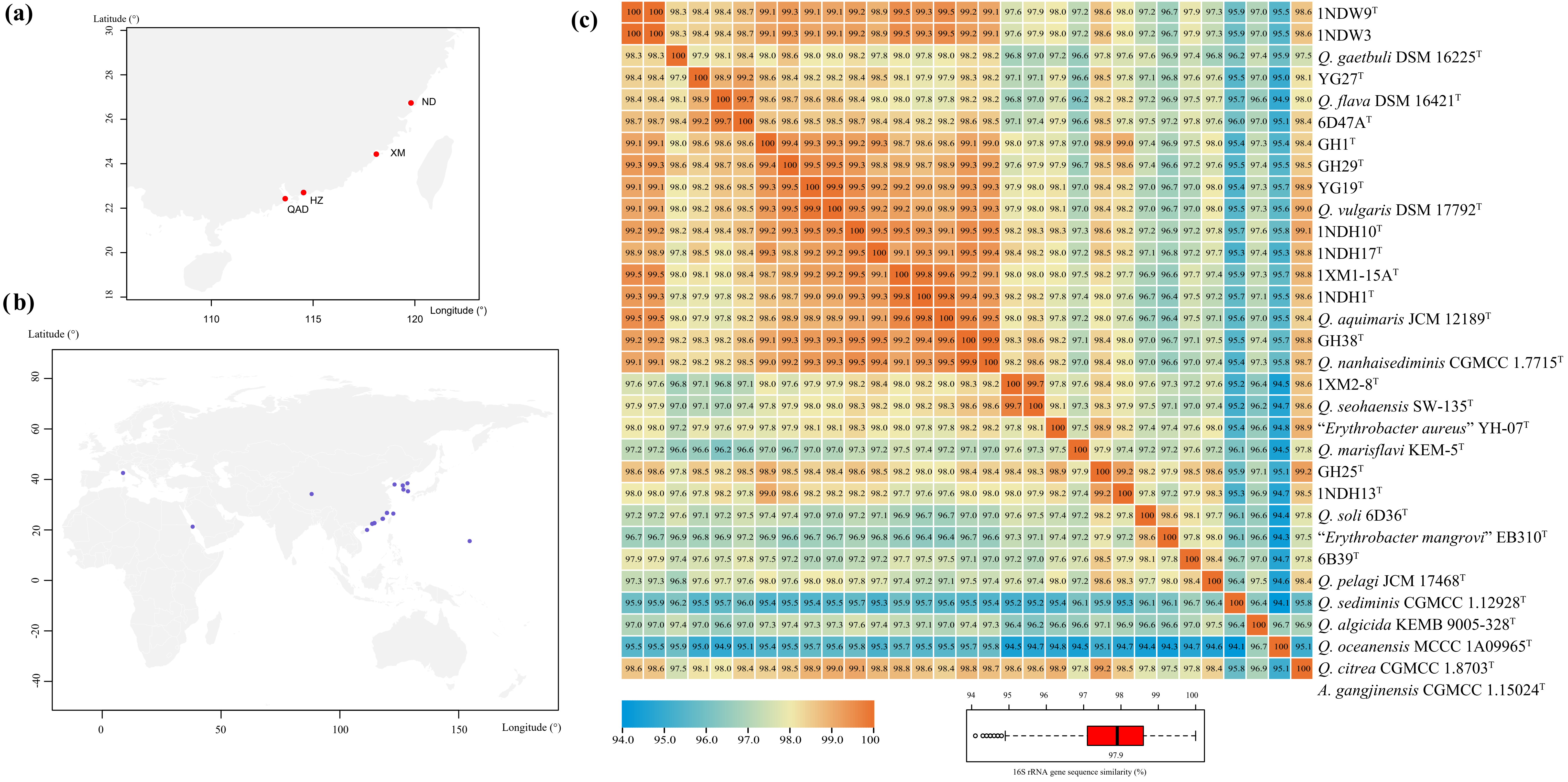

Figure S1 The sites distribution and the 16S RNA gene sequence-based analysis

(a) The four sampling sites indicated by red solid circles. (b) The isolated sites of 16 isolates and 15 reference type strains indicated by blue solid circles. (c) The heatmap of pairwise similarity matrix and the boxplot of similarity values of 16S rRNA gene sequences among all strains.

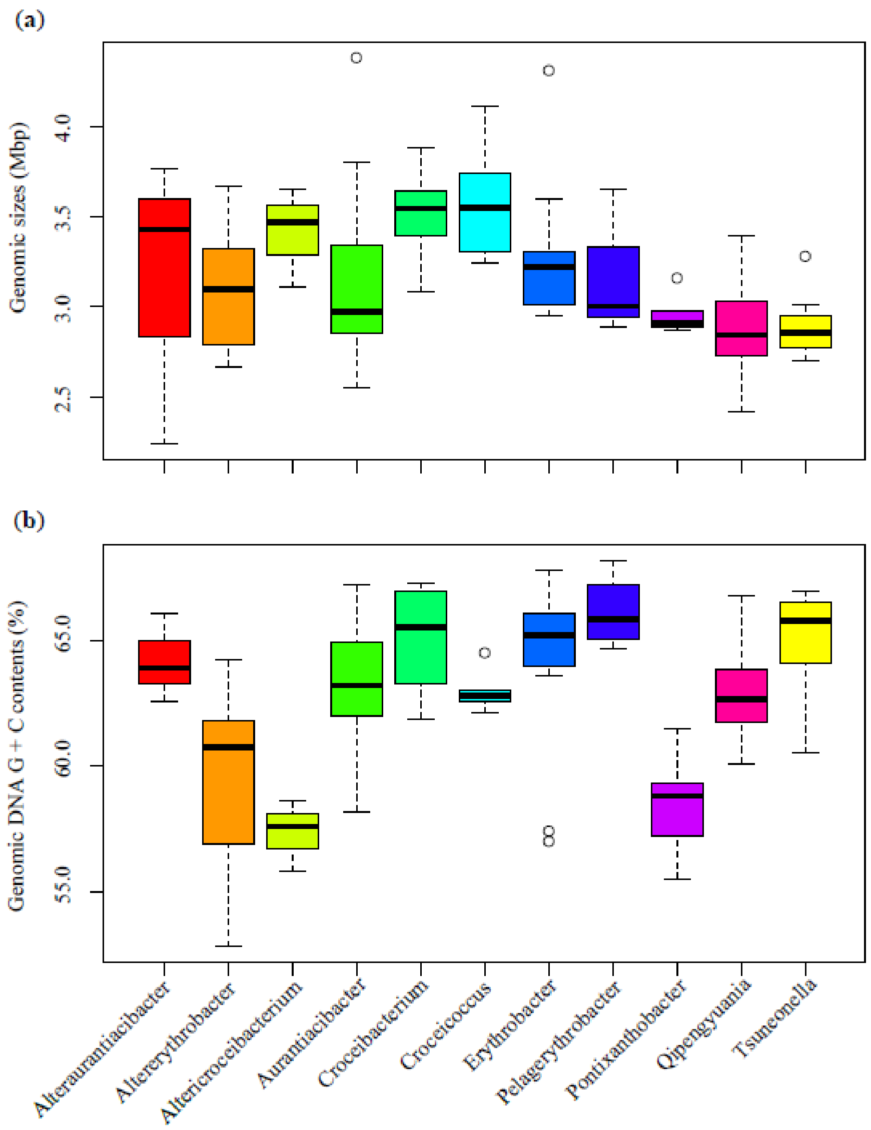

Figure S2 The genomic sizes (a) and DNA G + C contents (b) of the 11 genera within the family *Erythrobacteraceae*

Figure S3

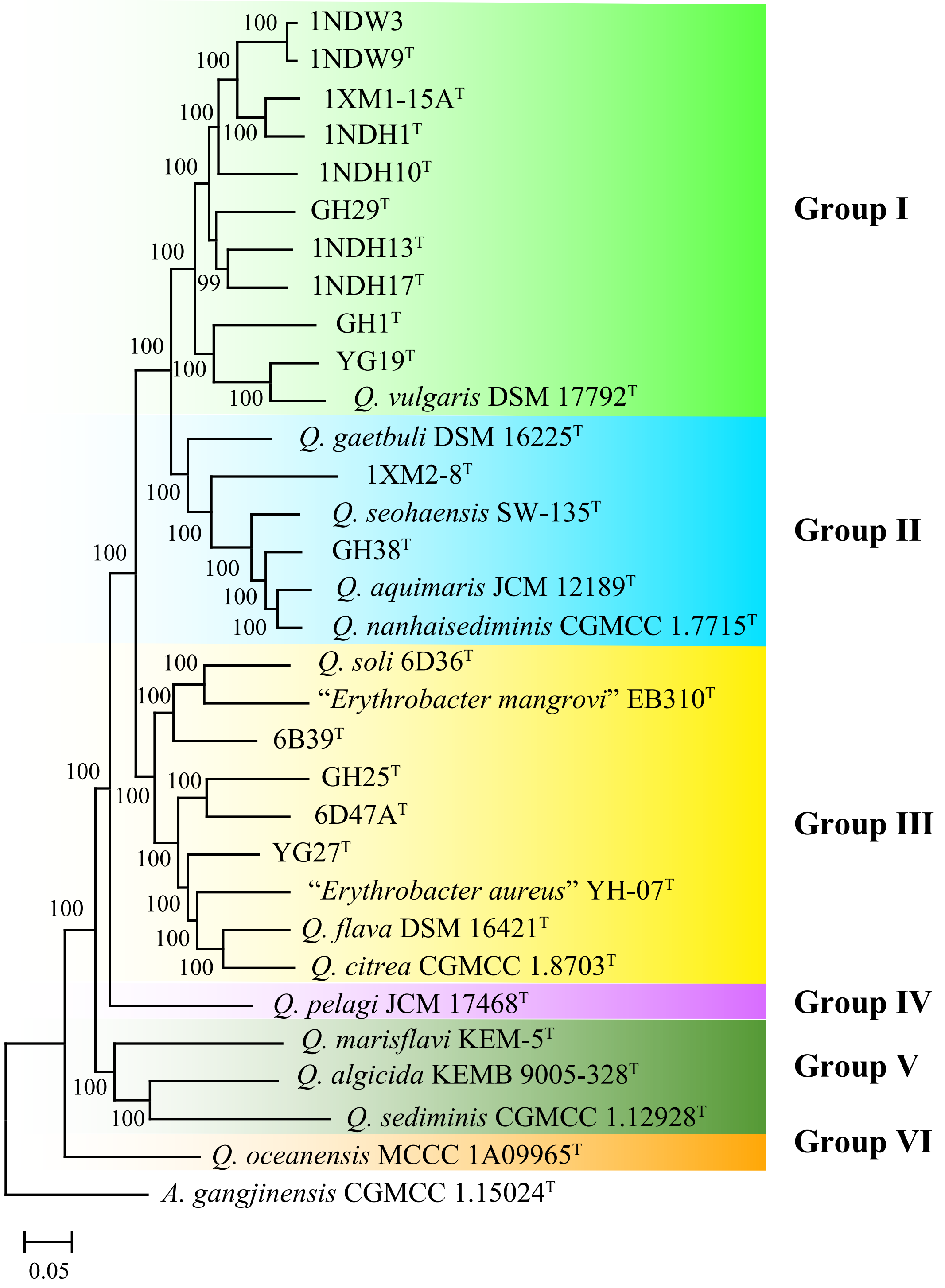
1NDW3
1NDW9T
1XM1-15AT
1NDH1T
1NDH10T
GH29T
1NDH13T
1NDH17T
GH1T
YG19T
Q. vulgaris DSM 17792T
Q. gaetbuli DSM 16225T
1XM2-8T
Q. seohaensis SW-135T
GH38T
Q. aquimaris JCM 12189T
Q. nanhaisediminis CGMCC 1.7715T
Q. soli 6D36T
"Erythrobacter mangrovi" EB310T
6B39T
GH25T
6D47AT
YG27T
"Erythrobacter aureus" YH-07T
Q. flava DSM 16421T
Q. citrea CGMCC 1.8703T
Q. pelagi JCM 17468T
Q. marisflavi KEM-5T
Q. algicida KEMB 9005-328T
Q. sediminis CGMCC 1.12928T
Q. oceanensis MCCC 1A09965T
A. gangjinensis CGMCC 1.15024T
Group I
Group II
Group III
Group IV
Group V
Group VI
100
99
0.05

Figure S3 Phylogenomic tree of bacteria within the genus *Qipengyuania* respectively based on bacterial core genes. The tree is inferred using the maximum likelihood method. Type strain *A. gangjinensis* CGMCC 1.15024^{T} (accession no. CP018097) is used as an outgroup. The species names are effectively but not yet validly published and thus are in quotation marks. Bootstrap values great than 80% are shown at branch points. Bar, 0.05 represents the number of substitutions per site.

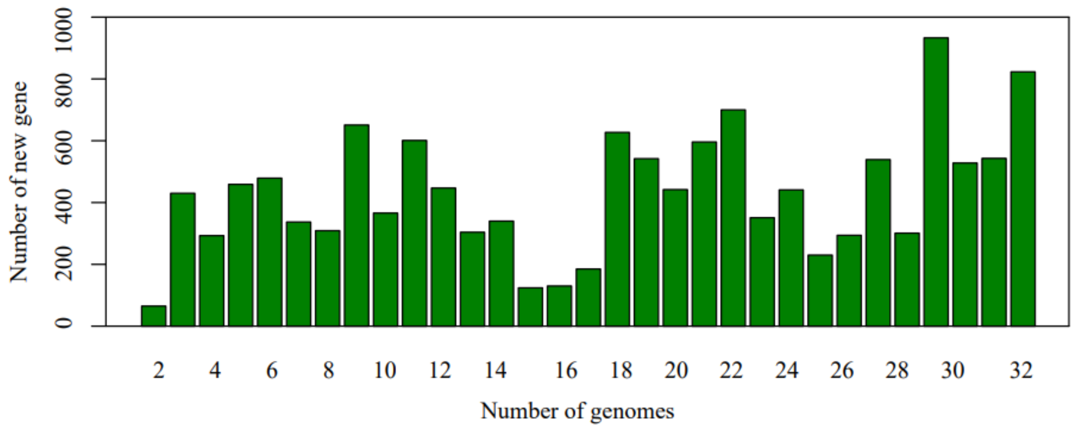

Figure S4 The number of new gene as the number of genomes increases

0 2 4 6 8 10

Strain	GT51	GT2	GH31	GH3	GT4	GH102	GH23	GT28	GT39	CE1	GT20	GH32	GH68	CE10	GH5	GH9	CBM48	GH13	GH51	GH18	GH43	GT25	GT26	GH30	GH65	GH94	GH95	GT2	GT84
1NDW9^T	1	1	1	1	1	1	1	1	1	1	1	1	1	0	0	0	0	0	0	0	0	0	0	0	0	0	0	0	0
1NDW3	4	3	2	1	2	1	1	1	1	1	1	1	1	0	0	0	0	0	0	0	0	0	0	0	0	0	0	0	0
1XM1-15A^T	4	4	2	1	1	1	1	1	1	1	1	0	0	0	0	0	1	1	0	0	0	0	0	0	0	0	0	0	0
1NDH1^T	4	3	2	1	2	1	1	1	1	1	1	1	1	0	0	0	1	1	0	0	0	0	0	0	0	0	0	0	0
1NDH10^T	4	3	2	1	1	1	1	1	1	1	1	0	0	0	0	0	0	0	0	0	0	1	0	0	0	0	0	0	0
GH29^T	4	2	2	2	2	1	1	1	1	1	1	0	0	0	2	2	0	0	0	0	0	0	0	0	0	0	0	0	0
1NDH13^T	4	2	2	1	1	1	1	1	1	1	1	0	0	0	0	0	0	0	0	0	0	0	0	0	0	0	0	0	0
1NDH17^T	4	3	2	1	1	1	1	1	1	1	1	0	0	0	0	0	1	1	0	0	0	0	0	0	0	0	0	0	0
GH1^T	4	2	3	5	0	1	1	1	1	1	1	0	0	0	1	1	0	0	1	0	1	0	0	0	0	0	0	0	0
YG19^T	4	3	2	1	0	1	1	1	1	1	1	0	0	0	0	0	0	0	0	0	0	0	0	0	0	0	0	0	0
Q. vulgaris DSM 17792^T	4	2	2	2	1	1	1	1	1	1	1	0	0	0	0	0	0	0	0	0	0	0	0	0	0	0	0	0	0
Q. gaetbuli DSM 16225^T	4	2	2	1	1	1	1	1	1	1	1	1	1	0	0	0	0	0	0	0	0	0	0	0	0	0	0	0	0
1XM2-8^T	4	2	2	1	1	1	1	1	1	1	1	0	0	0	0	0	0	0	0	0	0	0	0	0	0	1	0	0	1
Q. seohaensis SW-135^T	4	5	2	1	1	1	1	1	1	1	1	1	1	0	0	0	0	0	0	0	0	0	0	0	0	0	0	0	0
GH38^T	4	2	2	1	2	1	1	1	1	1	1	1	1	0	0	0	0	0	0	0	0	0	0	0	0	0	0	0	0
Q. aquimaris JCM 12189^T	4	3	2	1	2	1	1	1	1	1	1	1	1	0	0	0	1	1	0	0	0	0	1	0	0	0	0	0	0
Q. nanhaisediminis CGMCC 1.7715^T	4	2	2	1	1	1	1	1	1	1	1	0	0	0	0	0	1	1	0	0	0	0	0	0	0	0	0	0	0
Q. soli 6D36^T	4	3	3	2	3	1	1	1	1	1	0	0	0	0	0	0	0	0	0	0	0	0	1	0	0	0	0	0	0
"*Erythrobacter mangrovi*" EB310^T	4	3	2	2	2	1	1	1	1	1	0	0	0	1	0	0	0	0	0	0	0	0	0	0	1	0	0	0	0
6B39^T	4	4	3	2	1	1	1	1	1	1	1	0	0	0	1	1	0	0	1	0	1	0	0	0	0	0	0	0	0
GH25^T	4	3	1	2	1	1	1	1	1	1	1	0	0	2	1	1	0	0	0	1	0	0	0	0	0	0	0	1	0
6D47A^T	4	10	3	2	2	1	1	1	1	1	1	1	1	0	1	1	0	0	0	1	0	0	0	0	0	0	0	0	0
YG27^T	4	2	3	2	1	1	1	1	1	1	1	0	0	0	0	0	0	0	0	0	0	0	0	0	0	0	0	0	0
"*Erythrobacter aureus*" YH-07^T	4	2	0	1	0	1	1	1	1	1	1	0	0	0	0	0	0	0	0	0	0	0	0	0	0	0	0	0	0
Q. flava DSM 16421^T	4	4	2	1	0	1	1	1	1	1	1	0	0	1	0	0	0	0	0	0	0	0	0	0	0	0	0	0	0
Q. citrea CGMCC 1.8703^T	4	2	0	1	0	1	1	1	1	1	1	1	1	1	0	0	0	0	0	0	0	0	0	0	0	0	0	0	0
Q. pelagi JCM 17468^T	4	5	5	2	0	1	1	1	1	1	1	1	1	0	1	1	0	0	0	1	0	0	0	0	0	0	0	0	0
Q. marisflavi KEM-5^T	4	2	0	3	1	1	1	1	1	1	1	0	0	0	1	1	0	0	0	0	0	0	0	0	0	0	0	0	0
Q. algicida KEMB 9005-328^T	4	2	3	5	1	1	1	1	1	1	1	1	0	4	1	1	0	0	3	0	1	1	0	1	0	0	1	0	0
Q. sediminis CGMCC 1.12928^T	4	2	0	0	2	1	1	1	1	0	0	0	0	0	0	0	0	0	0	0	0	0	0	0	0	0	0	0	0
Q. oceanensis MCCC 1A09965^T	4	2	0	0	2	1	1	1	1	1	1	0	0	0	0	0	0	0	0	0	0	0	0	0	0	0	0	0	0

Figure S5 The number and distribution of genes encoding CAZymes in 31 strains

GT, glycosyltransferase; GH, glycoside hydrolase; CE, carbohydrate esterase; CBM, carbohydrate-binding molecule.

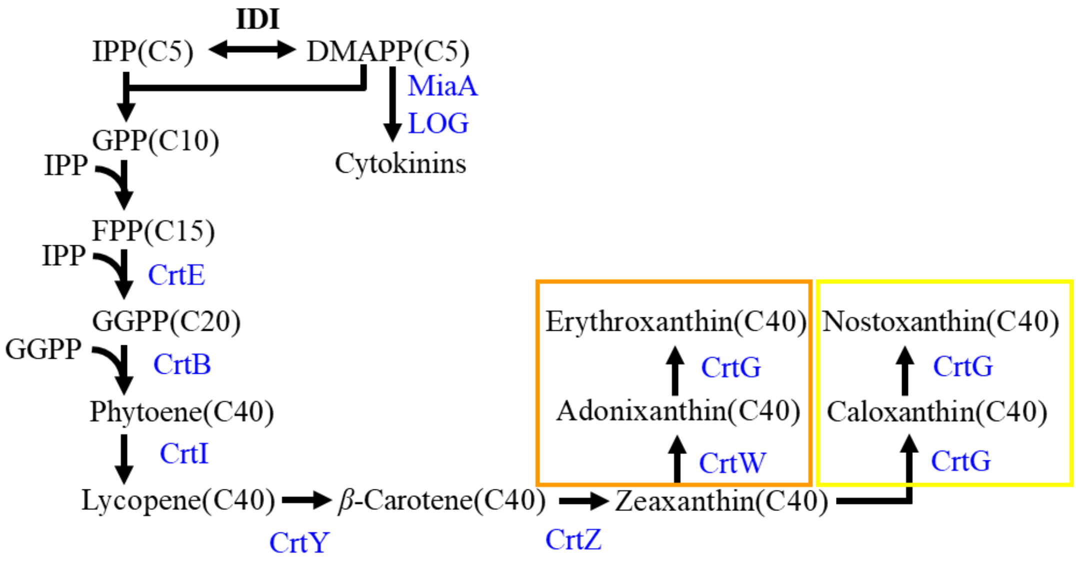

Figure S6 The presumptive pathway of carotenoid biosynthesis of the genus *Qipengyuania*

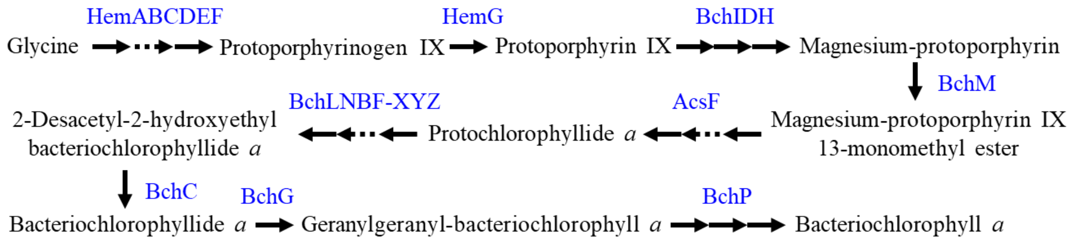

Figure S7 The presumptive bacteriochlorophyll *a* biosynthesis pathway of strains 6B39^{T} and 6D47A^{T}

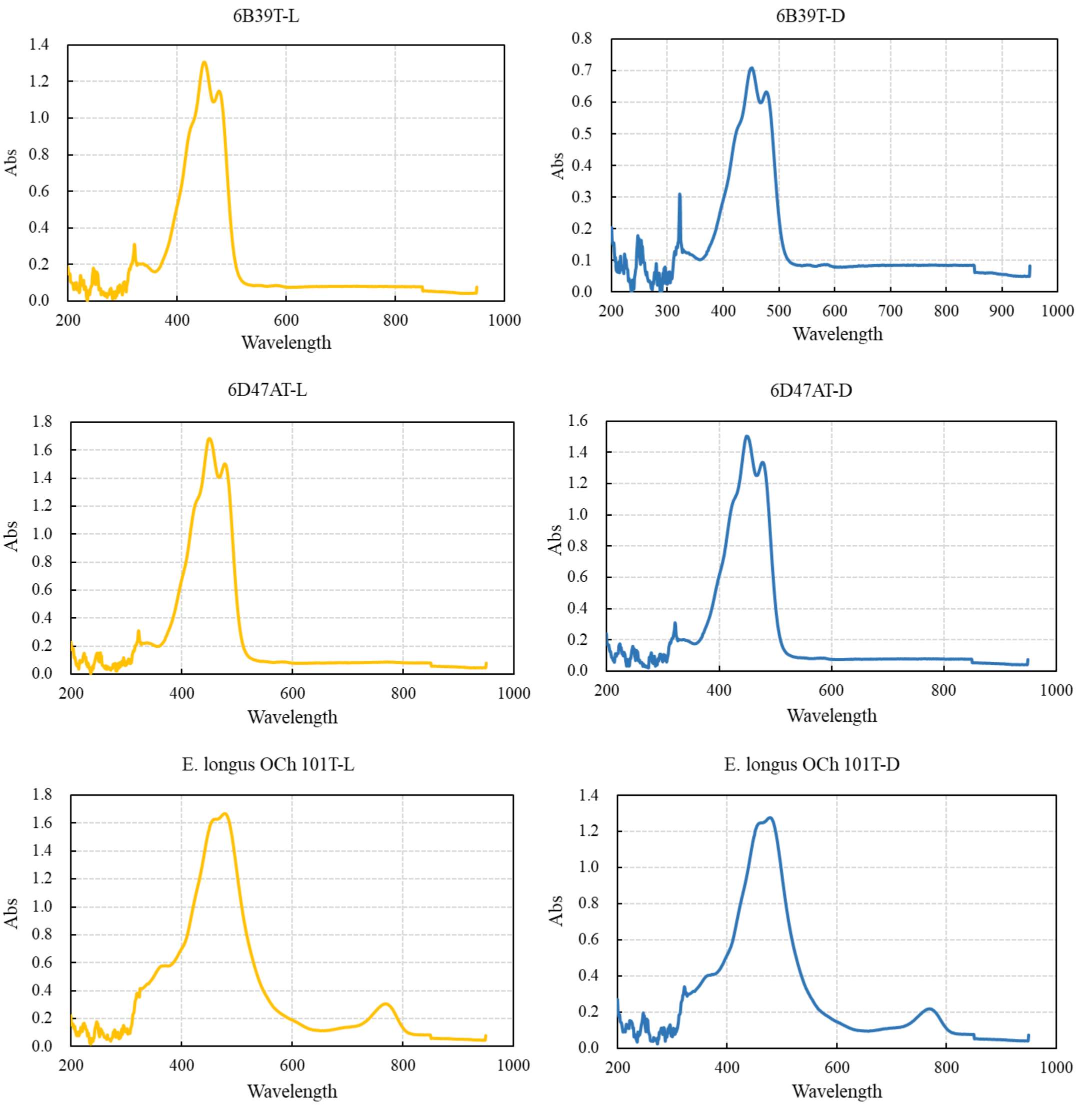

Figure S8 The full wavelength absorption spectra of the three strains under light (L) and dark (D) conditions

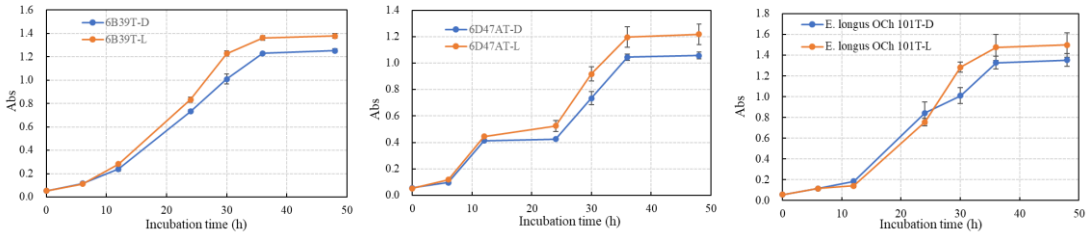

Figure S9 The growth curves of the three strains under light (L) and dark (D) conditions

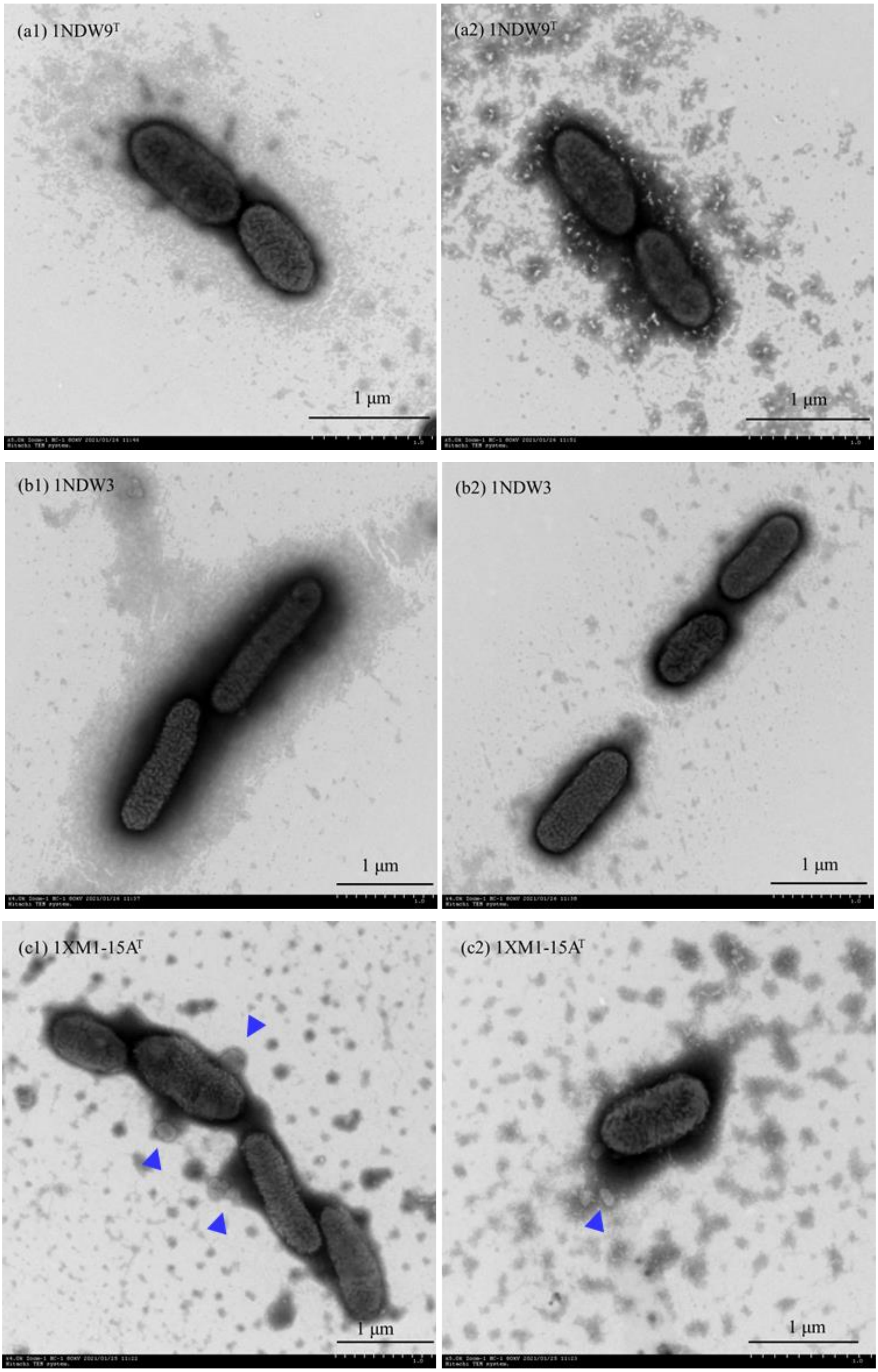
(a1) 1NDW9T
1 μm
(a2) 1NDW9T
1 μm
(b1) 1NDW3
1 μm
(b2) 1NDW3
1 μm
(c1) 1XM1-15AT
1 μm
(c2) 1XM1-15AT
1 μm

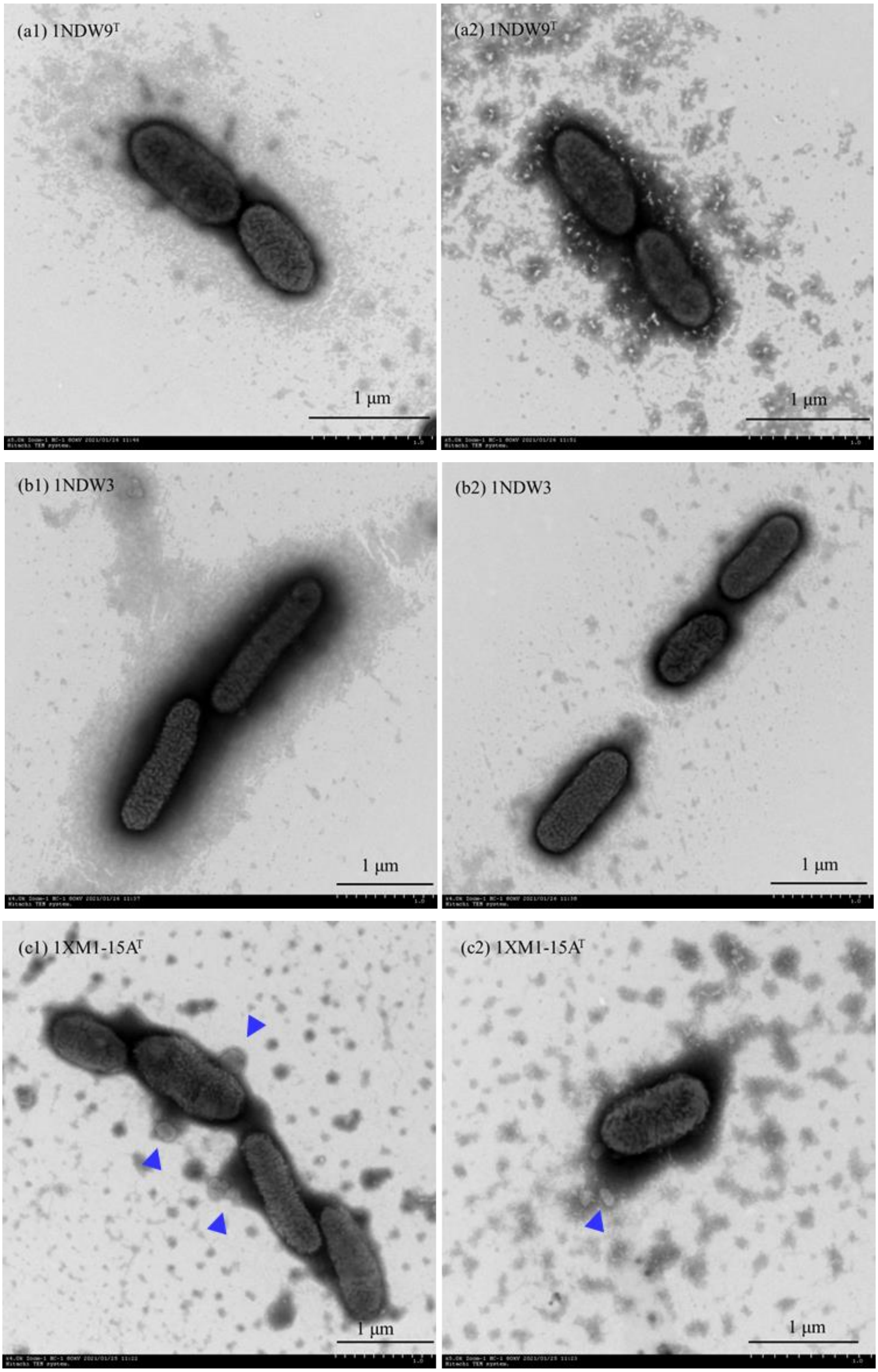
(a1) 1NDW9T
1 μm
(a2) 1NDW9T
1 μm
(b1) 1NDW3
1 μm
(b2) 1NDW3
1 μm
(c1) 1XM1-15AT
1 μm
(c2) 1XM1-15AT
1 μm

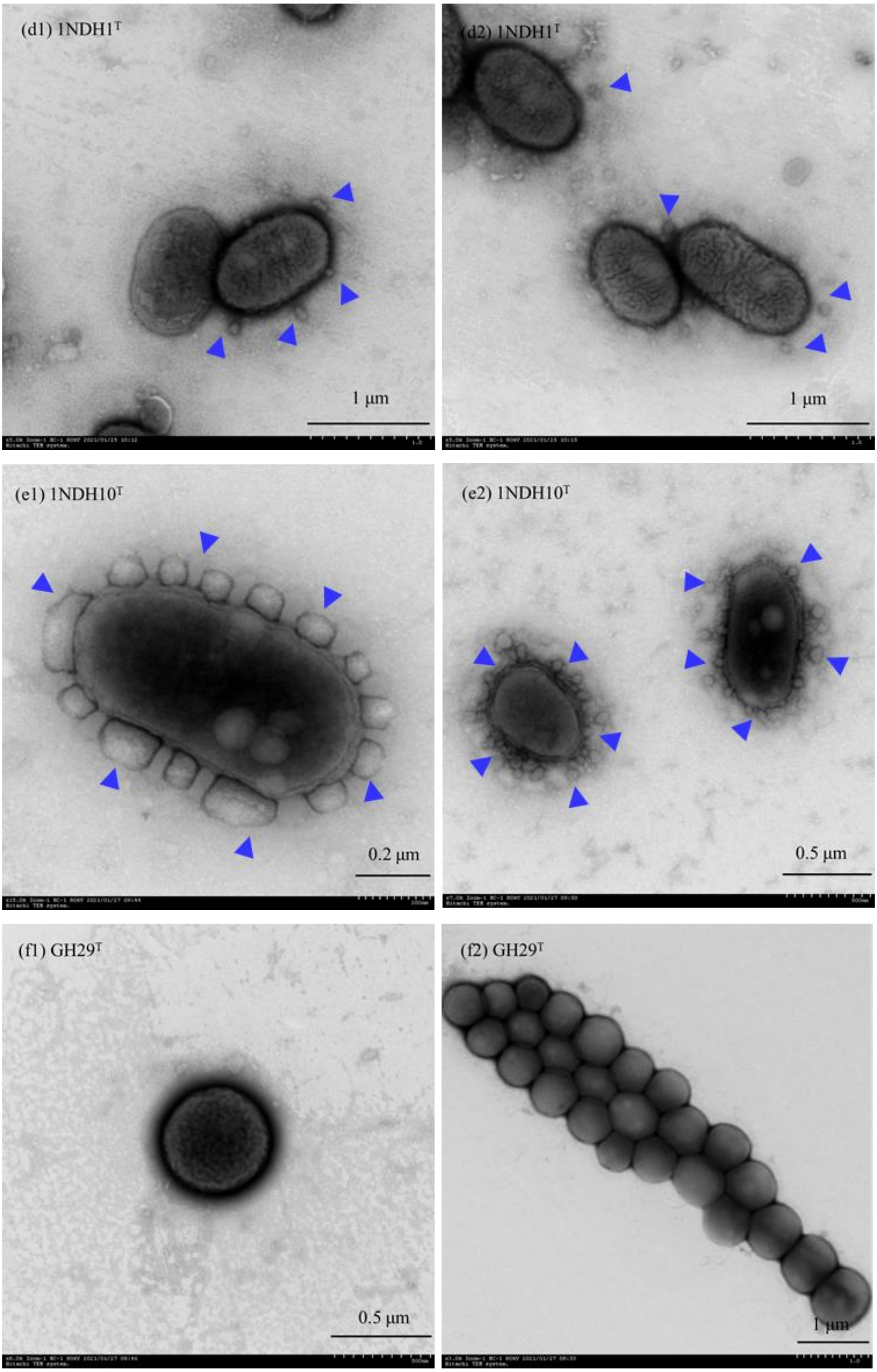
(d1) 1NDH1^{T}
1 µm
(d2) 1NDH1^{T}
1 µm
(e1) 1NDH10^{T}
0.2 µm
(e2) 1NDH10^{T}
0.5 µm
(f1) GH29^{T}
0.5 µm
(f2) GH29^{T}
1 µm

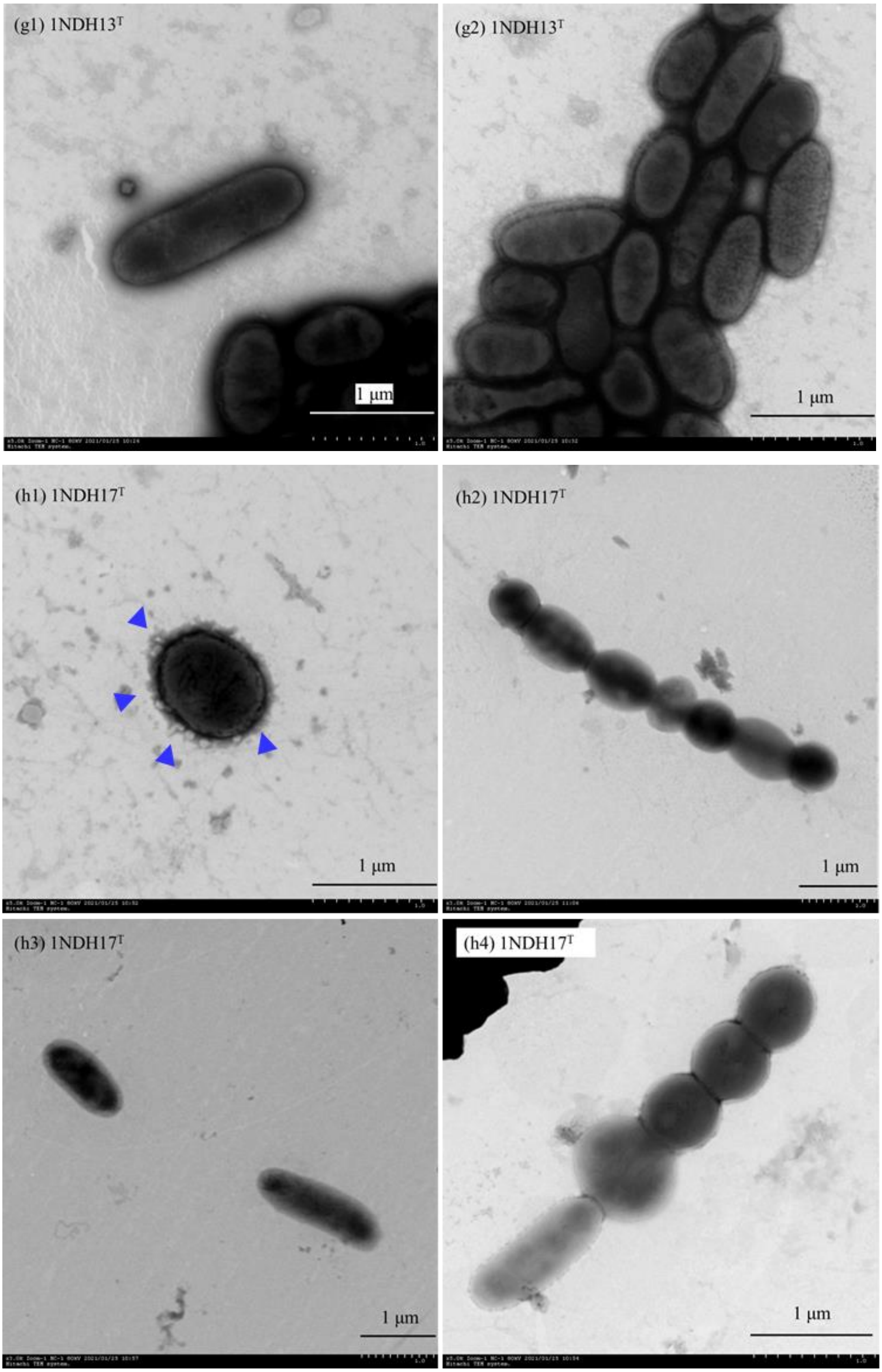
(g1) 1NDH13T
1 µm
(g2) 1NDH13T
1 µm
(h1) 1NDH17T
1 µm
(h2) 1NDH17T
1 µm
(h3) 1NDH17T
1 µm
(h4) 1NDH17T
1 µm

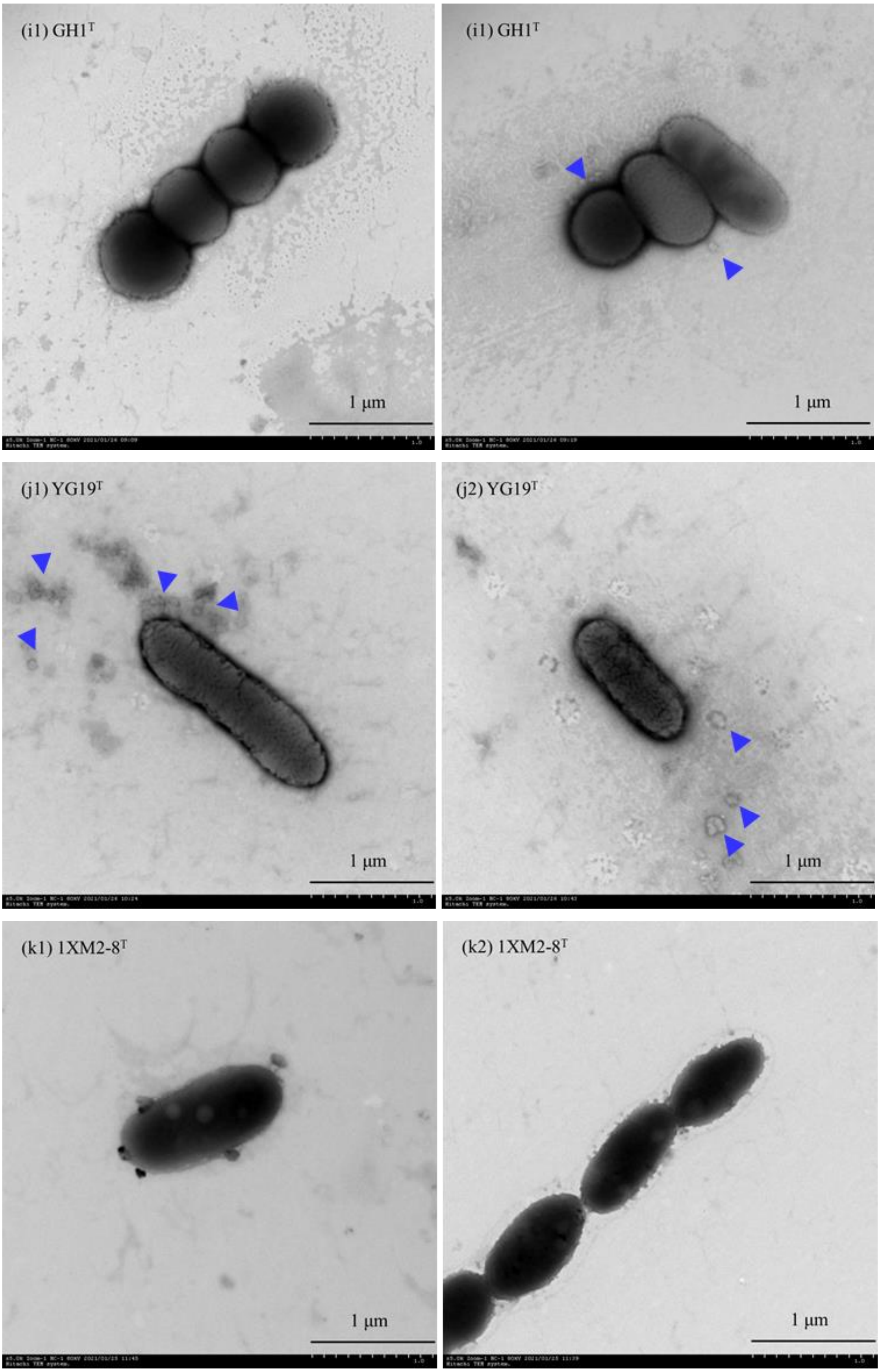
(i1) GH1T
1 µm
(i1) GH1T
1 µm
(j1) YG19T
1 µm
(j2) YG19T
1 µm
(k1) 1XM2-8T
1 µm
(k2) 1XM2-8T
1 µm

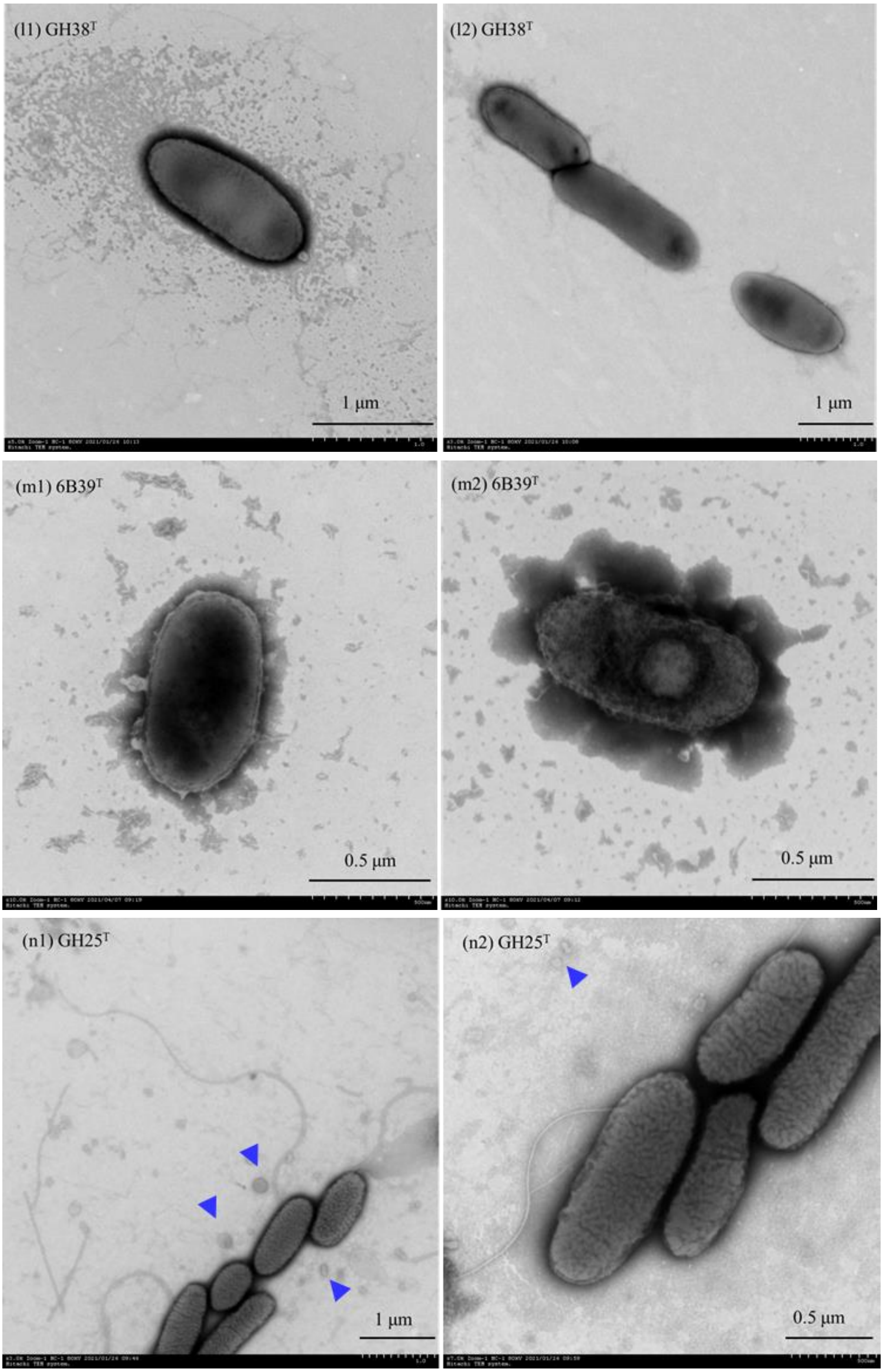
(l1) GH38T
1 µm
(l2) GH38T
1 µm
(m1) 6B39T
0.5 µm
(m2) 6B39T
0.5 µm
(n1) GH25T
1 µm
(n2) GH25T
0.5 µm

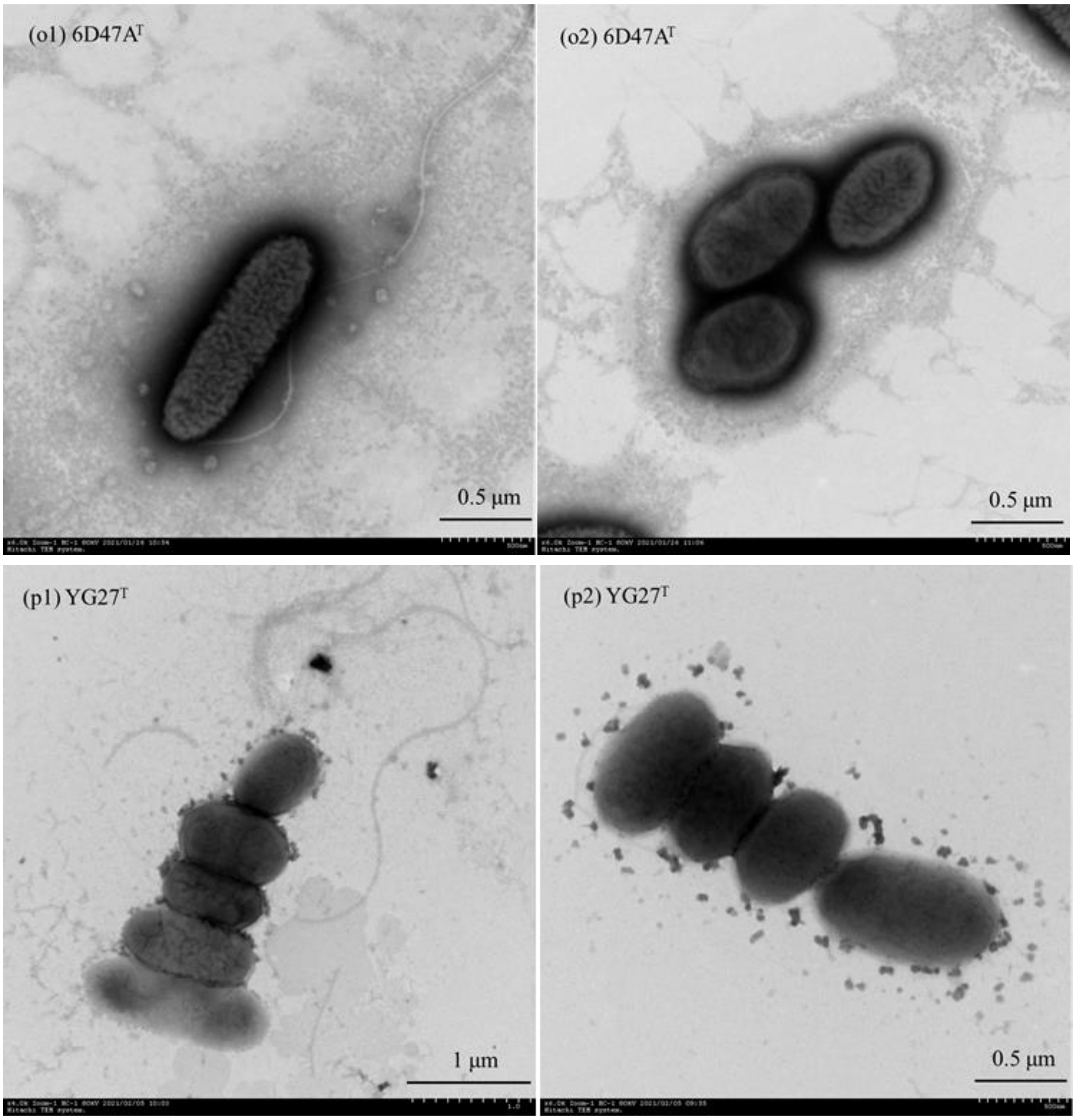

Figure S10 Morphological characteristics of cells of sixteen isolates grown on MA at 28 °C for 48 h.

The potential outer membrane vesicles or their analogues were indicated by blue solid triangles. a1-a2, 1NDW9^T; b1-b2, 1NDW3; c1-c2, 1XM1-15A^T; d1-d2, 1NDH1^T; e1-e2, 1NDH10^T; f1-f2, GH29^T; g1-g2, 1NDH13^T; h1-h4, 1NDH17^T; i1-i2, GH1^T; j1-j2, YG19^T; k1-k2, 1XM2-8^T; l1-l2, GH8^T; m1-m2, 6B39^T; n1-n2, GH25^T; o1-o2, 6D47A^T; p1-p2, YG27^T.

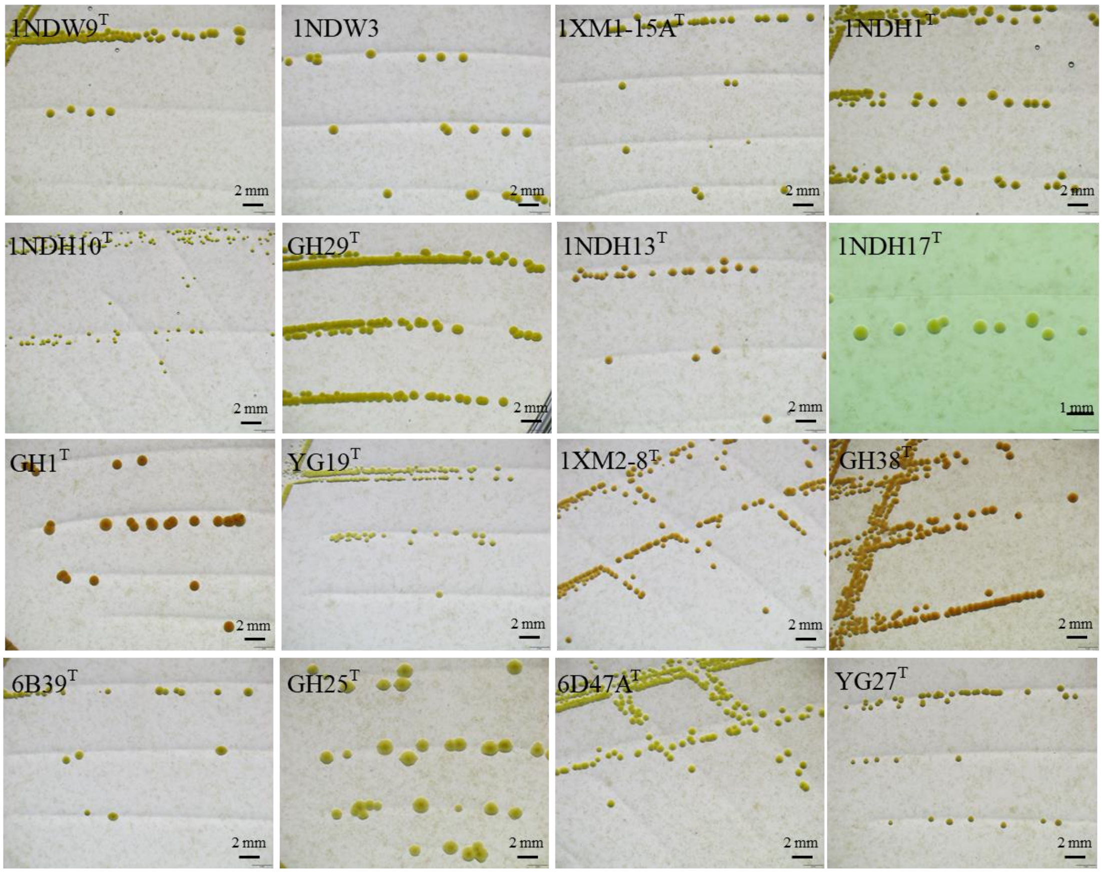

Figure S11 Morphological characteristics of colonies of sixteen isolates grown on MA at 28 °C for 48 h.

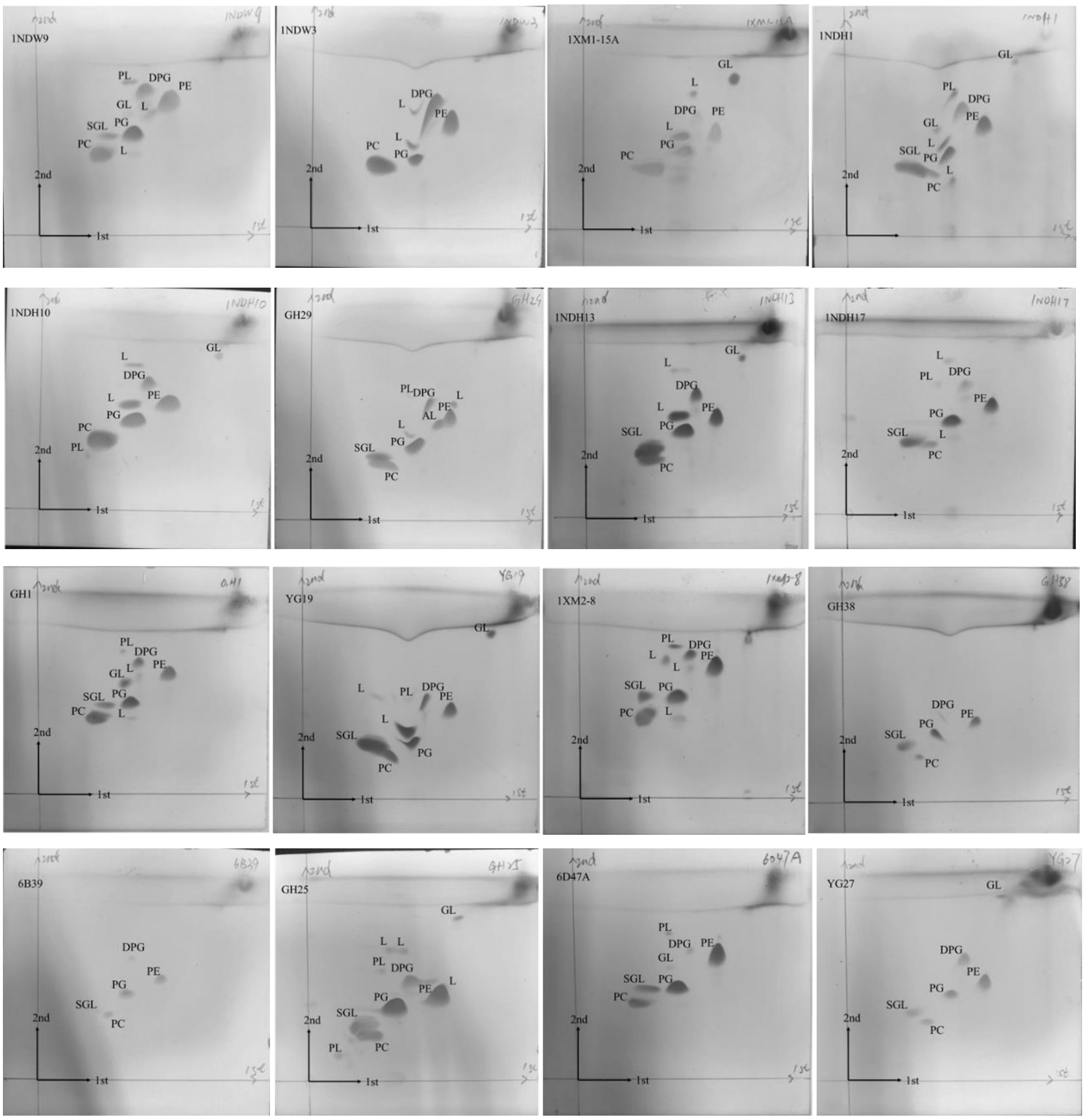

Figure S12 Polar lipids profiles of sixteen isolates

Abbreviations: DPG, diphosphatidylglycerol; PC, phosphatidylcholine; PE, phosphatidylethanolamine; PG, phosphatidylglycerol; AL, aminolipid; GL, glycolipid; PL, phospholipid; L, lipid; 1st, first dimension of TLC; 2nd, second dimension of TLC.

Table S1 General genomic characteristics of sixteen isolates and reference type strains

Strains	Accession No.	Size (Mbp)	DNA G + C content (%)	N50 (Mbp)	The number of				Quality (%)	
					Gene	CDS	tRNA	rRNA	Completeness	Contamination
1NDW9^T	JAIGNL000000000	2.71	63.5	1.82	2701	2650	47	3	99.5	0.5
1NDW3	CP081296	2.65	63.7	2.65	2654	2604	46	3	99.8	0.3
1XM1-15A^T	JAIGNM000000000	2.80	62.6	1.64	2774	2725	45	3	99.6	0.7
1NDH1^T	CP081294	2.83	62.7	2.83	2801	2751	46	3	99.5	0.9
1NDH10^T	JAIGNJ000000000	2.71	62.5	0.69	2692	2641	47	3	99.5	0.7
GH29^T	JAIGNR000000000	2.83	62.7	1.54	2814	2764	46	3	99.6	0.2
1NDH13^T	CP081295	2.83	63.8	2.83	2751	2692	51	6	99.8	0.0
1NDH17^T	JAIGNK000000000	2.85	63.3	0.70	2808	2759	45	3	99.5	0.0
GH1^T	JAIGNP000000000	2.93	61.1	0.93	2807	2757	46	3	99.5	0.7
YG19^T	JAIGNT000000000	2.55	60.6	0.18	2569	2516	46	6	99.2	0
Q. vulgaris DSM 17792^T	WTYC00000000	3.23	60.6	0.37	3198	3147	47	3	98.9	0.0
Q. gaetbuli DSM 16225^T	WTYF00000000	2.78	64.1	2.29	2772	2720	48	3	99.8	0.3
1XM2-8^T	CP081297	2.74	60.1	2.74	2699	2646	46	6	99.8	0.3
Q. seohaensis SW-135^T	CP024920	2.94	61.7	2.94	2929	2881	44	3	100	0
GH38^T	JAIGNS000000000	2.70	62.5	1.13	2659	2606	48	4	99.3	0.0
Q. aquimaris JCM 12189^T	WTYI00000000	2.66	61.8	2.66	2644	2593	47	3	99.7	0.0
Q. nanhaisediminis CGMCC 1.7715^T	FOWZ00000000	2.90	62.0	0.67	2890	2841	45	3	99.8	0.1
Q. soli 6D36^T	CP064654	2.91	63.3	2.91	2901	2850	47	3	99.7	0
"*Erythrobacter mangrovi*" EB310^T	CP053921	3.06	62.9	3.06	3035	2981	50	3	100	0
6B39^T	JAIGNN000000000	3.11	65.0	1.83	3052	3001	47	3	99.6	0.1
GH25^T	JAIGNQ000000000	3.17	60.7	0.93	3081	3025	49	6	99.9	0.3
6D47A^T	JAIGNO000000000	3.39	62.0	0.32	3377	3325	47	4	99.3	1.0

YG27^T	JAIGNU000000000	3.14	65.0	1.91	3068	3008	52	6	99.8	0.6
"*Erythrobacter aureus*" YH-07^T	CP031357	2.96	62.3	2.96	2970	2911	52	6	100	0
Q. flava DSM 16421^T	JAASQU000000000	2.81	63.9	0.76	2752	2700	48	3	99.8	0.2
Q. citrea CGMCC 1.8703^T	WTYG01000000	3.03	64.2	0.94	2948	2882	53	12	99.8	0.4
Q. pelagi JCM 17468^T	WTYD00000000	3.03	64.2	2.19	2931	2882	45	3	99.5	0
Q. marisflavi KEM-5^T	VCAO00000000	2.67	61.7	0.48	2623	2575	44	3	99.9	0
Q. algicida KEMB 9005-328^T	WTYA00000000	3.22	60.7	0.26	3109	3060	45	3	99.9	1.0
Q. sediminis CGMCC 1.12928^T	CP037948	2.42	66.8	2.42	2378	2326	48	3	100	0
Q. oceanensis MCCC 1A09965^T	WTYN00000000	2.87	63.9	2.17	2828	2776	48	3	99.2	0.2

Table S2 The 31 *Qipengyuania* strains used in this study

Strains	Accession No.*	Samples	Sources	Locations#	Isolated Media
1NDW9^T	MZ753461	Aquaculture pond sediment	Xiapu, Ningde, China	26.7341, 119.8089	MA
1NDW3	MZ749491	Aquaculture pond sediment	Xiapu, Ningde, China	26.7341, 119.8089	MA
1XM1-15A^T	MZ749493	Tidal flat sediment	Xiamen, China	24.4309, 118.1046	MA
1NDH1^T	MZ749487	Tidal flat sediment	Xiapu, Ningde, China	26.7341, 119.8089	MA
1NDH10^T	MZ749488	Tidal flat sediment	Xiapu, Ningde, China	26.7341, 119.8089	MA
GH29^T	MZ749498	Tidal flat sediment	Huizhou, China	22.7042, 114.5319	R2A
1NDH13^T	MZ749489	Tidal flat sediment	Xiapu, Ningde, China	26.7341, 119.8089	MA
1NDH17^T	MZ749490	Tidal flat sediment	Xiapu, Ningde, China	26.7341, 119.8089	MA
GH1^T	MZ749496	Tidal flat sediment	Huizhou, China	22.7042, 114.5319	R2A
YG19^T	MZ749500	Tidal flat sediment	Huizhou, China	22.7042, 114.5319	Improved R2A
Q. vulgaris DSM 17792^T	AY706935	Starfish *Stellaster equestris*	South China Sea	26.4717, 122.4833	MA
Q. gaetbuli DSM 16225^T	AY562220	Intertidal sediment	Yellow Sea, Korea	37.5922, 126.4568	MA
1XM2-8^T	MZ749492	Tidal flat sediment	Xiamen, China	24.4309, 118.1046	MA
Q. seohaensis SW-135^T	AY562219	Intertidal sediment	Yellow Sea, Korea	37.5922, 126.4568	MA
GH38^T	MZ749499	Tidal flat sediment	Huizhou, China	22.7042, 114.5319	R2A
Q. aquimaris JCM 12189^T	AY461441	Intertidal sediment	Yellow Sea, Korea	37.5922, 126.4568	MA
Q. nanhaisediminis CGMCC 1.7715^T	FJ654473	Marine sediment	South China Sea	20.0015, 111.4263	MA
Q. soli 6D36^T	MW186480	Mangrove soil	Qi'ao Island, China	22.4269, 113.6283	0.1*MA
"*Erythrobacter mangrovi*" EB310^T	MT522623	Mangrove rhizosphere soil	Zhangzhou, China	24.4, 117.95	MA
6B39^T	MZ749495	Mangrove soil	Qi'ao Island, China	22.4269, 113.6283	0.1*MA
GH25^T	MZ749497	Tidal flat sediment	Huizhou, China	22.7042, 114.5319	R2A
6D47A^T	MZ749494	Mangrove soil	Qi'ao Island, China	22.4269, 113.6283	0.1*MA

YG27^{T}	MZ749486	Tidal flat sediment	Huizhou, China	22.7042, 114.5319	Improved R2A
"*Erythrobacter aureus*" YH-07^{T}	MG756655	Maine sediment	Yellow Sea, China	Nd	Nd
Q. flava DSM 16421^{T}	AF500004	Seawater	East Sea, Korea	Nd	MA
Q. citrea CGMCC 1.8703^{T}	AF118020	Seawater	Mediterranean Sea, France	Nd	MA
Q. pelagi JCM 17468^{T}	HQ203045	Seawater	Red Sea	21.2829, 38.0498	MA
Q. marisflavi KEM-5^{T}	MK121890	Estuary water	Seocheon, Korea	36.0003, 126.7233	MA
Q. algicida KEMB 9005-328^{T}	KU981071	Seawater	Geoje Island, Korea	35.3073, 128.6508	MA
Q. sediminis CGMCC 1.12928^{T}	KJ734993	Subterrestrial sediment	Qiangtang Basin, China	34.2278, 88.1125	MA
Q. oceanensis MCCC 1A09965^{T}	KF924606	Deep-sea sediment	Western Pacific Oecan	15.5783, 154.6067	MA

*, the accession numbers of 16S rRNA gene sequences. #, latitude, longitude.

Table S3 Pan-genome distribution of 31 *Qipengyuania* genomes

Strains	Number of gene families		
	Core genes (%)	Accessory genes (%)	Unique genes (%)
1NDW9^T	1146 (43.2)	1428 (53.8)	64 (2.4)
1NDW3	1146 (44.0)	1426 (54.7)	22 (0.8)
1XM1-15A^T	1146 (42.0)	1445 (53.0)	120 (4.4)
1NDH1^T	1146 (41.7)	1439 (52.3)	148 (5.4)
1NDH10^T	1146 (43.4)	1291 (48.9)	191 (7.2)
GH29^T	1146 (41.5)	1384 (50.1)	218 (7.9)
1NDH13^T	1146 (42.6)	1350 (50.1)	166 (6.2)
1NDH17^T	1146 (41.5)	1400 (50.7)	201 (7.3)
GH1^T	1146 (41.5)	1257 (45.5)	332 (12.0)
YG19^T	1146 (45.5)	1152 (45.8)	191 (7.6)
Q. vulgaris DSM 17792^T	1146 (36.4)	1477 (46.9)	403 (12.8)
Q. gaetbuli DSM 16225^T	1146 (42.1)	1369 (50.3)	181 (6.7)
1XM2-8^T	1146 (43.3)	1259 (47.6)	228 (8.6)
Q. seohaensis SW-135^T	1146 (39. 8)	1512 (52.5)	193 (6.7)
GH38^T	1146 (44.0)	1353 (51.9)	90 (3.5)
Q. aquimaris JCM 12189^T	1146 (44.2)	1317 (50.8)	115 (4.4)
Q. nanhaisediminis CGMCC 1.7715^T	1146 (40.3)	1494 (52.6)	157 (5.5)
Q. soli 6D36^T	1146 (40.2)	1383 (48.5)	302 (10.6)
"*Erythrobacter mangrovi*" EB310^T	1146 (38.4)	1404 (47.1)	393 (13.2)
6B39^T	1146 (38.2)	1480 (49.3)	344 (11.5)
GH25^T	1146 (37.9)	1473 (48.7)	365 (12.1)
6D47A^T	1146 (34.4)	1536 (46.1)	578 (17.4)
YG27^T	1146 (38.1)	1555 (51.7)	264 (8.8)
"*Erythrobacter aureus*" YH-07^T	1146 (39.4)	1324 (45.5)	378 (13.0)
Q. flava DSM 16421^T	1146 (42.4)	1357 (50.2)	173 (6.4)
Q. citrea CGMCC 1.8703^T	1146 (39.7)	1371 (47.5)	255 (8.8)
Q. pelagi JCM 17468^T	1146 (39.8)	1191 (41.3)	471 (16.3)
Q. marisflavi KEM-5^T	1146 (44.5)	1103 (42.8)	278 (10.8)
Q. algicida KEMB 9005-328^T	1146 (37.4)	994 (32.5)	861 (28.1)
Q. sediminis CGMCC 1.12928^T	1146 (49.3)	643 (27.7)	518 (22.3)
Q. oceanensis MCCC 1A09965^T	1146 (41.3)	1046 (37.7)	543 (19.6)

Table S4 The proportion of core genes, accessory genes, and unique genes in different categories

Categories	The proportion (%)		
	Core genes	Accessory genes	Unique genes
Information storage and processing	22.6	11.0	15.7
Cellular processes and signaling	20.7	22.9	2.3
Metabolism	41.7	28.8	25.4
Poorly characterized	15.0	37.2	35.8

Table S5 The distribution of types of carotenoids biosynthesis cluster in 31 *Qipengyuania* strains

Types of carotenoids biosynthesis cluster*	Strains
I	1NDW9^T, 1NDW3, 1XM1-15A^T, 1NDH1^T, 1NDH10^T, *Q. vulgaris* DSM 17792^T, *Q. soli* 6D36^T, "*Erythrobacter mangrovi*" EB310^T, 6B39^T, 6D47A^T, YG27^T, "*Erythrobacter aureus*" YH-07^T, Q. flava DSM 16421^T, *Q. citrea* CGMCC 1.8703^T, *Q. sediminis* CGMCC 1.12928^T, *Q. oceanensis* MCCC 1A09965^T
II	GH29^T, 1NDH17^T, YG19^T, GH25^T, *Q. algicida* KEMB 9005-328^T
III	1NDH13T
IV	GH1^T, Q. gaetbuli DSM 16225^T, 1XM2-8^T, *Q. seohaensis* SW-135^T, GH38^T, *Q. aquimaris* JCM 12189^T, *Q. nanhaisediminis* CGMCC 1.7715^T, *Q. pelagi* JCM 17468^T, *Q. marisflavi* KEM-5^T

* The types of carotenoids biosynthesis cluster were defined, as shown in Figure 4a.

Table S6 Comparison of phenotypic characteristics between sixteen isolates and fifteen reference type strains

Characteristics	1NDW9^{T}	1NDW3	1XM1-15A^{T}	1NDH1^{T}	1NDH10^{T}	GH29^{T}	1NDH13^{T}	1NDH17^{T}	GH1^{T}	YG19^{T}	*Q. vulgaris* DSM 17792^{T}	*Q. gaetbuli* DSM 16225^{T}	1XM2-8^{T}	*Q. seohaensis* SW-135^{T}	GH38^{T}	*Q. aquimaris* JCM 12189^{T}	*Q. nanhaisediminis* CGMCC 1.7715^{T}	*Q. soli* 6D36^{T}	"*Erythrobacter mangrovi*" EB310^{T}	6B39^{T}	GH25^{T}	6D47A^{T}	YG27^{T}	"*Erythrobacter aureus*" YH-07^{T}	*Q. flava* DSM 16421^{T}	*Q. citrea* CGMCC 1.8703^{T}	*Q. pelagi* JCM 17468^{T}	*Q. marisflavi* KEM-5^{T}	*Q. algicida* KEMB 9005-328^{T}	*Q. sediminis* CGMCC 1.12928^{T}	*Q. oceanensis* MCCC 1A09965^{T}
**Cellular morphology**																															
Shape	Rods	Rods	Rods	Rods	Rods	Spherical	Rods	Pleomorphic	Pleomorphic	Rods	Rods	Rods	Rods	Rods	Rods	Rods	Rods	Rods	Rods	Short rods	Rods	Rods	Rods	Rods	Rods	Rods	Long rods	Pleomorphic	Pleomorphic	Ovoid rods	Ovoid or short rods
Length in size (μm)	0.8-1.1	0.8-1.6	0.9-1.4	0.8-1.0	0.6-0.9	0.6-0.9	1.2-2.4			1.2-2.0	1.2-1.8	2.0-4.0	0.6-1.5	1.5-4.0	1.0-2.0	2.0-4.0	0.5-1.7	1.1-1.3	0.8-1.6	0.5-0.8	0.6-1.0	0.6-1.1	0.8-1.4	1.1-1.5	1.5-2.5	1.0-1.5	2.5-3.0			0.5-1.2	0.7-1.7
Width in size (μm)	0.4-0.5	0.3-0.4	0.4-0.6	0.5-0.6	0.4-0.5		0.6-0.8			0.4-0.7	0.6-0.8	0.6-0.8	0.3-0.5	0.6-0.8	0.3-0.5	0.6-0.9	0.3-0.5	0.6-0.7	0.2-0.7	0.4-0.5	0.3-0.5	0.3-0.5	0.4-0.5	0.5-0.7	0.7-0.9	0.3-0.7	0.5-0.6			0.3-0.4	0.5-0.8
Motility	-	-	-	-	-	-	-	-	-	-	-	-	-	-	-	-	+	-	-	-	+	+	+	+	+	-	-	-	+	-	-
Outer membrane vesicles	-	w	+	+	+	-	+	+	+	+	nd	nd	-	nd	-	nd	nd	+	nd	-	+	-	-	nd	nd	nd	+	nd	nd	+	+
**Colony morphology**																															
Size (mm)	1	1	0.8	1	0.5	1.2	1	0.8	1	0.8	nd	1.0-2.0	0.8	1.0-2.0	0.8	1.0-2.0	1	1	0.5-1.5	1	1.6	0.8	0.6	1	1.0-1.5	nd	1.5-2.5	0.5-1.0	nd	nd	nd
Colour (Y, yellow; O, orange)	Y	Y	Y	Y	Y	Y	O	Y	O	Y	Y	Y-O	O	Y-O	O	O	O	Y	O	Y	Y	Y	Y	Y	Y	Y	O	Y-O	Y	Y	Y
**Growth conditions**																															
Temperature range	15-40	15-40	15-40	10-40	10-40	10-40	10-40	10-40	10-40	10-40	10-40	15-43	4-37	10-40	15-42	10-40	4-45	10-37	10-45	10-40	10-40	15-40	15-40	4-40	10-42	4-37	10-42	4-35	10-40	10-37	4-40
Optimal temperature	28	28	28	28	28	28	28	28	28	28	28-30	nd	28	nd	28	nd	nd	28	28-30	28	28	28	28	30	30-37	25-30	26-36	25	30	30	35-37
pH range	4-11	5-11	4-11	5-11	4-10	4-10	5-10	5-10	4-11	4-11	6-10	5.5-8	5-10	5.5-8	5-11	6-7.5	4.5-7.5	6.0-9.0	5.5-9.5	5-10	4-11	4-10	5-11	6.8-8.0	5.0-7.5	nd	6.0-9.0	5.5-8	4.0-9.0	6.5-9.5	6.0-10.0
Optimal pH	7	7	7	7-8	7	6-7	7	6-8	7-8	7	7.5-8.5	7-8	7	7-8	7-8	6.5-7.5	7.0-7.5	7	7.5	7	7-8	7	7	7	6.5-7.5	nd	8.0-9.0	7.0-8.0	9.0	8.0	7.0-8.0
NaCl range	0-10	0.5-8	0-10	2-7	0.5-8	0-10	0.5-7	2-6	0.5-7	0-10	1-8	1-9	2-7	1-9	0.5-7	1-10	1-10	0-8	0-9	2-9	0.5-7	0-9	0-10	1-7	1-13	nd	1-10	0.5-5	0-9	0-0.5	0-5.0
Optimal NaCl	3	3	1-2	3	3	2	3	3	3	2-3	1-3	2-3	3	2-3	3	2-3	2-3	3	2	3	3	3	3	3	2-2.5	nd	2-3	1.0-2.0	0	0	2.0-3.0
**Growth media**																															
MA	+	+	+	+	+	+	+	+	+	+	+	+	+	+	+	+	+	+	+	+	+	+	+	+	+	+	+	+	+	+	+
R2A	+	+	+	+	+	+	+	+	+	+	+	+	+	+	+	+	+	+	+	+	+	+	+	+	+	+	+	+	+	+	+
TSA	+	+w	+	-	+	+	-	-	+w	+	+	+	+	w	-	w	+	-	+	-	+	+w	+	+	+	-	+	+	+	+	-
NA	+	+	+	+	+	+	+	+	+	+	+	+	+	+	+	+	+	+	+	+	+	+	+	+	+	+	+	+	-	+	w
LB	+	+	+	+	+	+	+	+	+	+	+	+	+	+	+	+	+	+	+	+w	+	+	+	+	+	+	+	+	+	+	+
**Hydrolysis of**																															
Skimmed milk	-	-	-	-	-	-	-	-	-	-	-	-	-	-	-	-	-	-	-	+	-	-	-	-	-	-	-	-	-	-	-
Starch	-	-	-	-	-	-	-	-	-	-	-	-	-	-	-	-	-	-	-	-	-	-	-	-	-	-	-	-	-	-	-
Carboxymethyl cellulose	-	-	-	-	-	-	-	-	-	-	-	-	-	-	-	-	-	-	-	-	-	-	-	-	-	-	-	-	-	-	-
Teween 20	+	+w	+w	+	+	+w	+	+w	+	+	+	+	+w	+	+w	+	+	+	+	+w	+	+	+	-	+	+	+	+	+	+	+
Teween 40	+	+	w	+	+	+	+	-	+	+	+	+	-	+	+	+	+	+	+	-	+	+	+	+	+	-	+	+	+	+	+
Teween 60	+	+	+	+	+	+	+	-	+	+	+	+	-	+	+	+	+	+	+	-	+	+	+	+	+	-	+	+	+	+	+
Teween 80	-	-	-	+	-	-	+	-	+	-	+	-	-	-	-	-	-	-	+	-	+	-	-	-	-	-	-	+	+	+	-
**API ZYM**																															
Alkaline phosphatase	+	+	+	+	+	+	+	+	+	+	+	+	+	+	+	+	+	+	+	+	+	+	+	+	+	+	+	+	+	+	+
Esterase (C4)	+	+	+	+	+	+	+	+	+	+	+	+	+	+	+	+	+	+	+	+	+	+	+	+	+	+	+	+	+	+	+
Esterase lipase (C8)	+	+	+	+	+	+	+	+	+	+	+	+	+	+	+	+	+	+	+	+	+	+	+	+	+	+	+	+	+	+	+
Lipase (C14)	+	+	+	+	+	+	+	+	+	w	+	+	+	+	w	+	+	+	-	+	+	+	+	+	+	+	+	+	+	+	+
Leucine arylamidase	+	+	+	+	+	+	+	+	+	+	+	+	+	+	+	+	+	+	+	+	+	+	+	+	+	+	+	+	+	+	+
Valine arylamidase	+	+	+	+	+	+	+	+	+	+	+	+	+	+	+	+	+	+	+	+	+	+	+	+	+	+	+	+	+	+	+
Cystine arylamidase	+	+	+	+	+	+	+	+	+	+	+	+	+	+	+	+	+	+	+	+	+	+	+	+	+	+	+	+	+	+	+
Trypsin	+	+	+	+	+	+	+	+	+	+	+	+	+	+	+	+	+	+	+	+	+	+	+	+	+	w	+	+	+	+	+
α-Chymotrypsin	+	+	+	+	+	+	+	+	+	+	w	+	+	+	+	+	+	+	+	+	+	+	+	+	+	-	+	-	+	+	+
Acid phosphatase	+	+	+	+	+	+	+	+	+	+	+	+	+	+	+	+	-	+	+	+	+	+	+	+	+	+	+	+	+	+	+
Naphthol-AS-BI-phosphohydrolase	+	w	+	+	w	+	+	w	+	+	+	+	+	+	w	+	w	+	+	+	+	+	+	+	+	+	+	+	+	+	+
α-Galactosidase	+	+	-	-	-	+	-	-	-	-	-	-	-	-	-	-	-	+	-	w	-	-	-	-	-	-	-	-	-	-	-
β-Galactosidase	+	+	-	-	+	+	-	-	+	-	-	-	+	+	-	+	-	+	-	+	-	-	-	-	-	-	-	+	-	+	+
β-Glucuronidase	-	-	-	-	-	w	-	-	+	-	-	-	-	-	-	-	-	w	-	w	-	-	-	-	-	-	-	-	-	-	-
α-Glucosidase	+	+	+	+	w	+	+	+	+	+	+	-	+	+	w	+	-	+	+	+	-	+	+	-	+	-	+	-	+	-	-
β-Glucosidase	+	+	+	+	+	+	+	+	+	+	+	+	+	+	+	+	+	+	+	+	+	+	+	-	-	+	+	+	+	+	+
N-acetyl-*β*-Glucosaminidase	-	-	-	-	-	-	-	-	-	-	-	-	-	-	-	-	-	-	-	-	-	-	-	-	-	-	-	-	-	-	-
α-Mannosidase	-	-	-	-	-	-	-	-	-	-	-	-	-	-	-	-	-	-	-	-	-	-	-	-	-	-	-	-	-	-	-
α-Fucosidase	-	-	-	-	-	-	-	-	-	-	-	-	-	-	-	-	-	-	-	-	-	-	-	-	-	-	-	-	-	-	-
**API 20NE**																															
Reduction of nitrate	-	-	-	-	-	-	-	-	-	-	-	-	-	-	-	-	-	+	+	-	-	-	+	+	-	+	-	-	-	-	-
Denitrification	-	-	-	-	-	-	-	-	-	-	-	-	-	-	-	-	-	-	+	-	-	-	-	-	-	-	-	-	-	-	-
Indole production	-	-	-	-	-	-	-	-	-	-	-	-	-	-	-	-	-	-	-	-	-	-	-	-	-	-	-	-	-	-	-
D-Glucose fermentation	-	-	-	-	-	-	-	-	-	-	-	-	-	-	-	-	-	-	-	-	-	-	-	-	-	-	-	-	-	-	-

Arginine dihydrolase	-	-	-	-	-	-	-	-	+	-	-	-	-	-	-	-	-	-	-	-	-	-	-	-	-	-	-	-	-	-	-
Urease	-	-	-	-	+	-	-	-	+	-	-	-	-	-	-	-	-	-	-	-	-	-	-	-	-	-	-	-	-	-	-
β-Glucosidase (aesculin hydrolysis)	+	+	+	+	+	+	+	+	+	+	+	+	+	+	+	+	+	w	+	+	+	+	+	-	-	w	+	+	+	+	-
Gelatin hydrolysis	-	-	-	+	+	+	-	-	-	-	-	-	+	-	-	-	-	-	-	+	-	-	-	-	-	-	-	-	+	+	+
β-Galactosidase	+	+	-	-	+	+	-	-	+	-	-	-	+	+	-	+	-	+	-	+	-	-	-	-	-	-	-	+	-	+	+
D-Glucose	w	+	+	+	-	-	+	+	+	+	-	+	-	+	+	+	+	+	+	-	+	+	+	-	-	-	+	-	+	-	-
L-Arabinose	-	-	-	-	-	-	-	-	-	-	-	-	-	-	-	-	-	-	-	-	-	-	-	-	-	-	-	-	+	-	-
D-Mannose	-	-	-	-	-	-	-	-	-	-	-	-	+	-	-	-	-	-	-	-	w	-	-	-	-	-	-	-	+	-	-
D-Mannitol	-	-	-	-	-	-	-	-	-	-	-	-	-	-	-	-	-	-	-	-	-	-	-	-	-	-	-	-	-	-	-
N-acetyl-Glucosamine	-	-	-	-	+	-	-	-	-	-	-	-	-	-	-	-	-	-	-	-	-	-	-	-	-	-	-	-	-	-	-
D-Maltose	+	+	+	+	-	-	+	+	+	+	+	+	-	+	+	+	+	+	+	-	-	+	+	-	+	-	+	+	+	+	-
Potassium gluconate	-	-	-	-	-	-	-	-	-	-	w	-	-	-	-	-	-	-	-	-	+	-	+	-	+	-	-	-	-	-	w
Capric acid	-	-	-	-	-	-	-	-	-	-	-	-	w	-	-	-	-	-	-	+	-	-	-	-	-	-	-	-	-	-	-
Adipic acid	+	w	+	+w	w	-	-	+	+	w	w	-	-	-	+	+	+	w	+	-	+	w	+	w	+	+	w	+	w	-	-
Malic acid	-	-	-	+	+	-	-	-	-	+	-	-	-	-	w	+	+	w	-	-	+	-	+	-	-	w	+	-	+	+	-
Trisodium citrate	-	-	-	-	-	-	-	-	+	-	-	-	-	-	-	-	-	-	-	-	-	-	-	-	-	-	-	-	-	-	-
Phenylacetic acid	-	-	-	-	+	-	-	-	-	-	-	-	-	-	-	-	-	-	-	-	-	-	-	-	-	-	-	-	-	-	-
**Polar lipids**																															
DPG	+	+	+	+	+	+	+	+	+	+	+	nd	+	+	+	+	+	+	+	+	+	+	+	+	nd	+	+	+	+	+	+
PC	+	+	+	+	+	+	+	+	+	+	+	nd	+	+	+	+	+	+	+	+	+	+	+	+	nd	+	+	+	+	+	+
PE	+	+	+	+	+	+	+	+	+	+	+	nd	+	+	+	+	+	+	+	+	+	+	+	+	nd	+	+	+	+	+	+
PG	+	+	+	+	+	+	+	+	+	+	+	nd	+	+	+	+	+	+	+	+	+	+	+	+	nd	+	+	+	+	+	+
SGL	+	-	-	+	-	+	+	+	+	+	-	nd	+	-	+	-	-	+	+	+	+	+	+	+	nd	-	-	+	-	+	+
AL	-	-	-	-	-	+	-	-	-	-	-	nd	-	-	-	-	-	-	-	-	-	-	-	-	nd	-	-	-	-	-	+
GL	+	-	+	+, 2	+	-	+	+	+	+	-	nd	-	-	-	-	-	+	-	-	+	+	+	-	nd	-	-	-	-	+, 3	+, 2
PL	+	-	-	+	+	+	-	-	+	+	+	nd	+	+	-	+	-	+	-	-	+, 2	+	-	-	nd	-	-	+	+	+	+, 3
L	+, 2	+, 2	+, 2	+, 2	+	+, 2	+, 2	+, 2	+, 2	+, 2	-	nd	+, 3	+	-	+	+, 2	+	+, 5	-	+, 3	-	-	+, 2	nd	+	+, 2	+, 3	+, 3	+	+, 3

All data were obtained from this study, unless otherwise specified. +, positive or present; w, weakly positive; -, negative or absent; nd, no data. DPG, diphosphatidylglycerol; PC, phosphatidylcholine; PE, phosphatidylethanolamine; PG, phosphatidylglycerol; AL, aminolipid; GL, glycolipid; PL, phospholipid; L, lipid. Cellular and colony morphology, and growth conditions of reference type strains were obtained from the species descriptions in previous studies [1-9]. The data on polar lipids for type strains *Q. vulgaris* DSM 17792^T, *Q. seohaensis* SW-135^T, *Q. aquimaris* JCM 12189^T, *Q. nanhaisediminis* CGMCC 1.7715^T, and *Q. citrea* CGMCC 1.8703^T was obtained from the study by Xu *et al*. [1], that for type strain "*Erythrobacter mangrovi*" EB310^T was obtained from the study by Ye *et al*. [2], that for type strain *Q. algicida* KEMB 9005-328^T was obtained from the study by Kristyanto *et al*. [3], that for type strain *Q. oceanensis* MCCC 1A09965^T was obtained from the study by Yang *et al*. [4], that for type strain *Q. sediminis* CGMCC 1.12928^T was obtained from the study by Feng *et al*. [5], that for type strain *Q. pelagi* JCM 17468^T was obtained from the study by Wu *et al*. [6], that for type strain "*Erythrobacter aureus*" YH-07^T was obtained from the study by Tang *et al*. [7], that for type strain *Q. marisflavi* KEM-5^T was obtained from the study by Park *et al*. [8], that for type strain *Q. soli* 6D36^T was obtained from the study by Liu *et al*. [9].

Table S7 Cellular fatty acid profiles of 16 isolates and 15 reference type strains

Fatty acids	1ND W9^T	1ND W3	1XM1-15A^T	1ND H1^T	1NDH 10^T	GH 29^T	1NDH 13^T	1NDH 17^T	GH 1^T	YG 19^T	*Q. vulgaris* DSM 17792^T	*Q. gaetbuli* DSM 16225^T	1XM2-8^T	*Q. seohaensis* SW-135^T	GH 38^T	*Q. aquimaris* JCM 12189^T	*Q. nanhaisediminis* CGMCC 1.7715^T	*Q. soli* 6D36^T	"*Erythrobacter mangrovi*" EB310^T	6B3 9^T	GH 25^T	6D47 A^T	YG 27^T	"*Erythrobacter aureus*" YH-07^T	*Q. flava* DSM 16421^T	*Q. citrea* CGMCC 1.8703^T	*Q. pelagi* JCM 17468^T	*Q. marisflavi* KEM-5^T	*Q. algicida* KEMB 9005-328^T	*Q. sediminis* CGMCC 1.12928^T	*Q. oceanensis* MCCC 1A09965^T
$C_{9:0}$	TR	TR	TR	TR	ND	TR	ND	TR	ND	TR	TR	TR	TR	ND	ND	ND	TR	TR	TR	TR	1.0	TR	TR	TR	ND	ND	TR	TR	TR	TR	TR
$C_{12:0}$	0.6	0.5	TR	TR	TR	0.8	0.7	0.6	0.7	TR	0.5	0.6	0.6	TR	TR	0.6	0.6	1.4	0.8	0.8	2.5	0.8	0.7	0.9	0.7	ND	0.8	0.8	0.8	1.3	1.1
$C_{14:0}$	TR	TR	TR	0.6	0.5	TR	TR	TR	0.8	TR	TR	TR	TR	0.6	TR	0.7	TR	TR	TR	TR	0.7	TR	TR	TR	TR	TR	TR	0.7	TR	0.9	TR
$C_{15:0}$	1.7	1.5	2.0	2.3	1.1	0.6	0.6	1.6	0.7	3.4	1.4	1.0	1.5	2.0	1.6	2.2	1.2	1.2	4.3	1.7	0.8	0.6	1.7	ND	0.5	TR	3.4	ND	ND	ND	0.6
$C_{16:0}$	10.6	9.9	12.2	11.8	13.8	9.5	3.5	11.4	6.3	8.4	9.2	9.1	8.5	12.5	11.2	11.0	10.3	7.3	4.4	7.4	9.7	11.6	6.8	6.2	7.6	3.2	5.5	9.1	7.0	15.4	4.4
$C_{17:0}$	2.7	2.0	2.4	1.3	0.5	1.3	TR	1.9	TR	4.8	1.9	0.9	1.0	0.7	1.2	0.8	0.7	1.5	2.7	2.1	ND	TR	1.4	0.5	0.8	0.6	3.8	2.5	2.4	3.9	TR
$C_{18:0}$	TR	TR	TR	TR	0.5	0.7	TR	0.5	TR	TR	TR	TR	TR	TR	TR	TR	TR	TR	TR	TR	ND	1.1	TR	0.6	TR	TR	TR	TR	0.7	0.6	TR
$C_{8:0}$ 3OH	ND	ND	ND	ND	ND	ND	ND	ND	ND	ND	ND	ND	ND	ND	ND	ND	ND	ND	ND	ND	1.1	ND	ND	ND	ND	TR	ND	ND	ND	ND	ND
$C_{14:0}$ 2OH	3.3	3.8	5.0	6.8	6.0	6.0	6.5	4.7	11.5	2.8	5.0	8.1	6.8	6.9	5.7	7.4	7.6	5.5	1.3	3.3	13.2	4.3	3.4	3.1	2.8	3.6	2.2	5.5	7.3	3.1	4.0
$C_{15:0}$ 2OH	7.8	8.1	7.1	5.9	1.9	5.9	7.6	5.5	1.2	14.0	5.6	3.2	5.2	3.6	4.5	3.7	3.5	5.6	9.3	5.3	3.1	0.6	5.6	2.2	2.7	0.7	6.5	8.1	6.3	5.5	2.4
$C_{16:0}$ 2OH	2.6	2.9	1.1	0.7	6.1	4.0	2.9	1.1	1.6	1.4	0.7	1.5	1.6	1.7	2.1	1.3	1.8	4.1	2.1	1.9	10.6	4.6	4.0	5.3	2.0	3.0	TR	0.8	TR	TR	2.6
$C_{17:0}$ 2OH	ND	TR	ND	TR	TR	0.6	TR	ND	ND	TR	ND	ND	ND	TR	TR	TR	TR	0.6	0.5	TR	ND	ND	TR	ND	ND	TR	ND	TR	ND	ND	ND
$C_{19:0}$ cyclo *ω*8*c*	ND	ND	ND	ND	TR	ND	ND	ND	ND	ND	ND	ND	ND	ND	ND	ND	ND	ND	ND	ND	ND	ND	ND	ND	ND	ND	ND	ND	1.1	ND	ND
anteiso-$C_{15:0}$	ND	ND	ND	2.7	ND	ND	ND	ND	ND	ND	ND	ND	ND	ND	ND	ND	ND	ND	ND	ND	ND	ND	ND	ND	ND	ND	ND	ND	TR	TR	ND
anteiso-$C_{17:0}$	ND	TR	TR	1.4	ND	ND	ND	ND	ND	ND	TR	TR	ND	ND	ND	ND	ND	TR	TR	ND	ND	ND	ND	TR	ND	TR	ND	ND	ND	TR	ND
iso-$C_{16:0}$	ND	ND	ND	1.6	ND	ND	ND	ND	ND	ND	ND	TR	ND	ND	ND	ND	ND	ND	ND	ND	ND	ND	ND	ND	ND	ND	TR	ND	ND	ND	ND
iso-$C_{18:0}$	ND	ND	ND	ND	ND	ND	ND	ND	ND	ND	ND	ND	ND	ND	ND	ND	ND	ND	ND	ND	ND	ND	ND	ND	ND	1.7	ND	ND	ND	ND	ND
iso-$C_{19:0}$	0.6	ND	ND	ND	ND	0.7	ND	0.7	ND	ND	TR	ND	ND	ND	ND	ND	ND	TR	ND	TR	ND	ND	ND	ND	ND	ND	ND	ND	ND	TR	ND
$C_{15:1}$ *ω*6*c*	ND	TR	TR	TR	TR	ND	ND	TR	TR	TR	ND	TR	ND	TR	TR	TR	ND	TR	0.9	TR	ND	TR	ND	ND	ND	ND	0.6	2.2	TR	TR	ND
$C_{16:1}$ 2OH	ND	ND	ND	ND	TR	ND	1.2	ND	TR	ND	0.9	ND	TR	TR	ND	TR	ND	ND	ND	ND	0.7	ND	ND	TR	TR	2.0	ND	ND	ND	ND	1.1
$C_{16:1}$ *ω*5*c*	TR	0.6	0.9	0.8	0.9	TR	ND	0.9	1.0	TR	TR	1.8	1.1	1.6	1.2	1.6	1.6	1.1	0.7	1.0	1.2	0.7	1.0	1.4	TR	2.3	TR	TR	1.1	0.8	2.3
$C_{17:1}$ *ω*6*c*	23.6	23.3	20.9	16.3	4.8	11.4	1.0	18.1	2.8	10.5	3.3	11.2	17.6	11.3	12.9	11.9	11.3	17.2	39.9	24.6	ND	2.6	20.2	7.8	1.0	4.9	39.9	19.3	20.4	31.9	11.8
$C_{17:1}$ *ω*8*c*	1.4	1.5	1.5	1.0	TR	0.6	3.3	1.0	TR	2.7	3.0	1.2	1.3	0.9	1.1	1.1	1.0	1.3	4.4	1.9	ND	ND	1.8	TR	1.6	TR	4.9	1.6	1.1	3.4	2.0
$C_{18:1}$ 2OH	TR	TR	TR	TR	TR	TR	1.9	ND	0.7	TR	0.8	0.7	0.5	TR	0.7	TR	0.8	0.9	TR	TR	1.5	TR	TR	0.8	0.9	1.8	0.5	TR	TR	ND	2.0
$C_{18:1}$ *ω*5*c*	0.7	0.8	0.9	0.5	0.8	0.8	ND	0.7	0.6	TR	TR	1.0	0.9	0.8	1.0	0.6	1.1	1.0	0.5	1.0	0.6	0.9	0.8	1.8	TR	1.7	0.6	0.6	1.0	TR	1.0
$C_{18:1}$ *ω*7*c* 11-methyl	ND	ND	ND	4.6	8.2	ND	9.1	6.9	11.4	8.8	ND	4.3	4.8	4.5	5.7	4.0	5.5	ND	ND	ND	4.3	4.3	ND	5.5	5.2	4.9	1.9	9.8	11.0	ND	ND
$C_{18:3}$ *ω*6*c* (6, 9, 12)	TR	TR	TR	TR	TR	TR	TR	TR	TR	TR	TR	TR	TR	TR	TR	TR	TR	0.5	TR	0.5	1.8	TR	0.5	TR	TR	TR	TR	TR	TR	TR	TR
Summed feature 3#	7.6	8.2	11.3	11.6	11.2	8.4	12.9	8.0	13.8	9.2	20.0	13.1	12.1	15.7	10.0	18.2	12.6	9.4	7.5	12.5	17.7	10.9	11.4	9.0	13.8	14.0	4.9	16.6	6.5	10.2	18.6
Summed feature 6#	ND	TR	ND	ND	0.5	ND	ND	ND	0.8	ND	ND	ND	ND	ND	0.5	ND	ND	ND	ND	ND	ND	ND	ND	ND	ND	TR	ND	ND	ND	ND	ND
Summed feature 7#	TR	TR	TR	TR	ND	TR	ND	TR	ND	TR	ND	TR	TR	TR	TR	ND	TR	TR	0.6	0.6	ND	ND	TR	TR	ND	TR	0.8	TR	ND	TR	TR
Summed feature 8#	33.4	32.3	30.6	25.8	41.0	45.8	46.5	33.6	44.1	30.8	45.4	38.8	33.0	34.2	37.8	32.8	37.7	36.7	15.7	32.1	29.3	54.3	37.8	51.6	58.6	51.9	20.7	19.4	29.9	19.5	42.6
Summed feature 9#	0.7	1.1	1.1	0.8	TR	0.9	ND	0.7	0.5	ND	ND	0.8	0.8	0.6	0.7	0.5	0.6	1.3	0.7	0.5	ND	0.6	TR	TR	ND	TR	0.7	ND	TR	0.7	1.1

All data was obtained from this study, unless otherwise specified. Values were percentages of total fatty acids. Less than 0.5% and/or the absence of fatty acids for all strains were not shown. The predominant cellular fatty acid for all strains was higer than 10%. TR, trace amount (< 0.5%); ND, not detected. #, summed features are fatty acids that cannot be resolved reliably from another fatty acid using the chromatographic conditions chosen. The MIDI system groups these fatty acids together as one feature with a single percentage of the total. Summed feature 3, $C_{16:1}$ *ω*7*c* and/or $C_{16:1}$ *ω*6*c*; summed feature 6, $C_{19:1}$ *ω*11*c* and/or $C_{19:1}$ *ω*9*c*; summed feature 7, $C_{19:1}$ *ω*7*c* and/or $C_{19:1}$ *ω*6*c*; summed feature 8, $C_{18:1}$ *ω*6*c* and/or $C_{18:1}$ *ω*7*c*; summed feature 9, $C_{16:0}$ 10-methyl and/or iso-$C_{17:1}$ *ω*9*c*. The data of fatty acids for type strain *Q. soli* 6D36^T was obtained from the study by Liu *et al*. [9].

REFERENCES

[1] Xu M, Xin Y, Yu Y, *et al*. *Erythrobacter nanhaisediminis* sp. nov., isolated from marine sediment of the South China Sea. International journal of systematic and evolutionary microbiology, 2010, 60(9): 2215-2220.

[2] Ye Y-H, Anwar N, Xamxidin M, *et al*. Description of *Erythrobacter mangrovi* sp. nov., an aerobic bacterium from rhizosphere soil of mangrove plant (*Kandelia candel*). Antonie van Leeuwenhoek, 2020, 113(10): 1425-1435.

[3] Kristyanto S, Lee S D, Kim J. *Porphyrobacter algicida* sp. nov., an algalytic bacterium isolated from seawater. International journal of systematic and evolutionary microbiology, 2017, 67(11): 4526-4533.

[4] Yang Y, Zhang G, Sun Z, *et al*. *Altererythrobacter oceanensis* sp. nov., isolated from the Western Pacific. Antonie van Leeuwenhoek, 2014, 106(6): 1191-1198.

[5] Feng X-M, Mo Y-X, Han L, et al. *Qipengyuania sediminis* gen. nov., sp. nov., a member of the family *Erythrobacteraceae* isolated from subterrestrial sediment. International journal of systematic and evolutionary microbiology, 2015, 65(10): 3658-3665.

[6] Wu H, Lai P Y, Lee O O, *et al*. *Erythrobacter pelagi* sp. nov., a member of the family *Erythrobacteraceae* isolated from the Red Sea. International journal of systematic and evolutionary microbiology, 2012, 62(6): 1348-1353.

[7] Tang T, Sun X, Dong Y, *et al*. *Erythrobacter aureus* sp. nov., a plant growth-promoting bacterium isolated from sediment in the Yellow Sea, China. 3 Biotech, 2019, 9(11): 1-9.

[8] Park S, Won S M, Yoon J H. *Erythrobacter marisflavi* sp. nov., isolated from isolated from estuary water. International journal of systematic and evolutionary microbiology, 2019, 69(9): 2696-2702.

[9] Liu Y, Pei T, Deng M-R, *et al*. *Qipengyuania soli* sp. nov., isolated from mangrove soil. Current microbiology, 2021: 1-9.
